# Anthropomorphism Unveiled: A Decade of Systematic Insights in Business and Technology Trends

**DOI:** 10.12688/f1000research.162157.1

**Published:** 2025-03-10

**Authors:** Diesyana Ajeng Pramesti, Budhi Haryanto, Lilik Wahyudi, Catur Sugiarto

**Affiliations:** 1 Department of Management, Faculty Economic and Business, Universitas Muhammadiyah Magelang, Magelang, Central Java, Indonesia; 2 Department of Management, Faculty Economic and Business, Universitas Sebelas Maret, Surakarta, Central Java, Indonesia

**Keywords:** Anthropomorphism, Systematic Literature Review, Bibliometric Analysis

## Abstract

Anthropomorphism studies have been conducted over the past decade; however, there is a void in the literature that provides an overview of anthropomorphism studies in business, management, and accounting. This article provides an in-depth analysis and mapping of major studies in the literature linked to anthropomorphism over the last 14 years by providing a topical classification consistent with present and future anthropomorphism research. The critical function of anthropomorphism in marketing communication tactics necessitates a thorough evaluation that is currently lacking, supplementing past studies to support academics’ and practitioners' interests in performing a thorough analysis of anthropomorphism in future marketing communication trends. This article summarizes studies on anthropomorphism in business, management, and accounting published in Scopus-indexed journals between 2010 and 2024 using Bibliometric-R and VOS viewer in compliance with the PRISMA protocol. The findings highlight significant trends in the articles, including the evolution of the literature (theories and methodologies employed), publications, authors, countries, journal performance, and trends in supporting research themes in the past, present, and future. Research on anthropomorphism has grown rapidly, particularly from 2022 to 2024. This paper provides a complete summary of the fragmented literature to guide future research.

## 1. Introduction

Anthropomorphism is a form of communication in which humans are represented by non-human entities and has become a significant phenomenon in the modern business world. In this era of rapidly evolving technology, anthropomorphism has emerged as a viable solution for improving communication between humans and non-human entities. Anthropomorphism can lead to more meaningful personal interactions ( Wan & Chen, 2021). It is a topic that can influence consumer perception, behaviour, and psychology. Merchants use this condition to increase customer purchases ( Guido & Peluso, 2015; J. Kim et al., 2018; Zhang et al., 2022). For example, the recent increase in the use of chatbots has resulted in a pleasant and welcoming tone and a speaking manner similar to that of humans. This fosters trust and emotional ties between the brand and its customers ( Aggarwal & McGill, 2012; Puzakova et al., 2013).

In the business world, anthropomorphism changes the focus from a functional viewpoint of brands or products to an emotional one from the customer’s perspective ( Puzakova & Aggarwal, 2018). Consumers who were initially interested in buying a product brand because of the functions attached to that brand, but with anthropomorphism, this can transform into an emotional closeness that influences them. Does the brand align with its own characteristics? Marketers use this gap to reach their desired target markets. In the field of management, anthropomorphism strategies have long-term implications, namely, strengthening relationships with stakeholders ( Lee & Oh, 2021) and increasing productivity ( Aggarwal & McGill, 2012). Companies can display an empathetic image through anthropomorphism that reflects values aligned with stakeholders to strengthen relationships ( Choi et al., 2021). Businesses can maintain stakeholder support even when the business environment undergoes dynamic changes ( Puzakova et al., 2013). Similarly, in accounting, anthropomorphism is used to present complex financial data to increase engagement ( Vorontsova, 2024). Through anthropomorphism, consumer understanding becomes easier, consumer trust in product brands increases, and it supports relevant transparency and accountability ( Wan & Chen, 2021). As evidenced by numerous university research groups that have started studying anthropomorphism, it has been combined with robotic AI technology in the past four years ( Troshani et al., 2021). This can significantly strengthen empathy, influence perceptions, and influence consumer preferences ( Choi et al., 2021).

With continuous technological development, the application of anthropomorphism has become increasingly challenging. However, this limitation cannot be ignored. If this strategy is used correctly, marketers can easily attract consumers, build consumer trust in the brand, and create consumer loyalty. However, if this anthropomorphism is used excessively or improperly, significant risks may arise, such as manipulative perceptions, distrust, and even the “uncanny valley” ( Mori et al., 2012). Therefore, a careful, ethical, effective, and relevant approach is required for target markets. Marketers must understand consumer psychology and emotions. This study provides a systematic review of the development of anthropomorphism over the past decade, particularly in the fields of business, management, and accounting ( Pranckutė, 2021). Through bibliometric analysis, this study aims to comprehensively explore and deeply assess the development of anthropomorphism in the past and present, related to the identification of themes, patterns of collaboration among authors, and the impact of published articles, particularly in the fields of business, management, and accounting. Additionally, this study aims to identify existing research gaps and provide recommendations for future studies. This study provides new insights into how anthropomorphism has become a transformative strategy in the face of modern business challenges.

To achieve that goal, this study has several research questions as follows:

1. What is the trend of research and publication on the topic of anthropomorphism?
2. How has the academic literature on anthropomorphism developed from 2010 to 2024, specifically regarding themes, theoretical foundations, and methodologies used?
3. How many papers have been published in different journals by authors, institutions, and their affiliated countries?
4. How will the direction of anthropomorphism research be in the future look?

These four research questions provide an overview of the evolution of anthropomorphism research in response to the emergence of newly connected authors, presenting new topics that become challenges and research opportunities with implications for business dynamics and corporate managerial decision-making. Bibliometric studies provide an overview and framework for anthropomorphism studies from their inception in 2010 to 2024 in the domains of business, management, and accounting. This period was chosen because anthropomorphism began to grow in 2010 and has been steadily increasing in publications until 2024. The objective of this study is to provide insight and valuable information on the issues and themes that receive the greatest attention and create niches in academia, which can then be exploited in future research. Furthermore, this literature evaluation can help marketers, marketing practitioners, business operators, and stakeholders optimize their strategies and make better managerial decisions.

## 2. Literature review

Anthropomorphism is a marketing communication strategy that uses non-human entities that possess characteristics, traits, and behaviours similar to those of humans ( Epley et al., 2007). The association between humans and nonhuman objects has both functional and emotional relevance. Anthropomorphism creates relationships and emotional connections between consumers and businesses. The perspective of anthropomorphism depends on an individual’s view of the environment and surroundings ( Fawcett, 1989). The perspective of anthropomorphism is influenced by an individual’s cultural background ( Spatola et al., 2022); thus, the way anthropomorphism is communicated is adjusted according to how consumers respond to it ( Baskentli et al., 2023). Brand anthropomorphism enhances consumer perception; therefore, marketers must position brands correctly because different brands have different perception capacities ( Lee & Oh, 2021). Using communication strategies such as AI-based anthropomorphism, a brand can be anthropomorphized without changing the form of the product ( F. R. Chen et al., 2021).

The implementation of the Technology Acceptance Model (TAM) and Unified Theory of Adoption and Use of Technology (UTAUT) for anthropomorphism is reflected in the use of chatbots with names, voices, language styles, or expressions that resemble humans, making them feel more intimate and closer to users ( Venkatesh et al., 2003). Naturally built emotional closeness fosters a level of trust and high commitment, similar to interacting with fellow humans ( Brown et al., 2019). Repeated interactions imply patterns of positive consistency and brand preference ( Seric et al., 2019). Anthropomorphism enriches consumer experience and plays a significant role in enhancing overall business performance. However, marketers must be smart in designing anthropomorphism in AI as naturally as possible because excessive and inauthentic anthropomorphism can trigger discomfort and distrust among consumers. Anthropomorphism can lead to a shift in the consumer mindset to focusing more on abstract or emotional rather than functional attributes ( Wan & Chen, 2021). Anthropomorphism is an effective strategy for building relationships with consumers and heavily supports the development of modern businesses that use technology.

## 3. Methods

Bibliometric studies have become important instruments for researchers who wish to examine and gain deeper insight into a particular field. Analysis of academic publications reveals research patterns, highlights leading researchers, and identifies areas that require further exploration. This process is crucial for understanding the current state of the research and guiding future research ( Zolfagharian et al., 2019). This study not only counts the number of articles, authors, and citations but also focuses on emerging fields and collaborative patterns among authors and institutions. Bibliometric studies allow researchers to handle large amounts of data without risking bias ( Donthu et al., 2021).

This research adopted the PRISMA (Preferred Reporting Items for Systematic Reviews and Meta-Analyses) protocol, which includes identification, screening, eligibility, and inclusion ( Hansen et al., 2022; Kuckertz & Block, 2021; Lim et al., 2022). PRISMA is conducted to ensure that the approach is systematic and unbiased by identifying and including high-quality studies, making it reliable for conclusions. Through this study, deeper insight into specific fields was obtained.

### 3.1 Bibliometric techniques used

**Figure 1.**
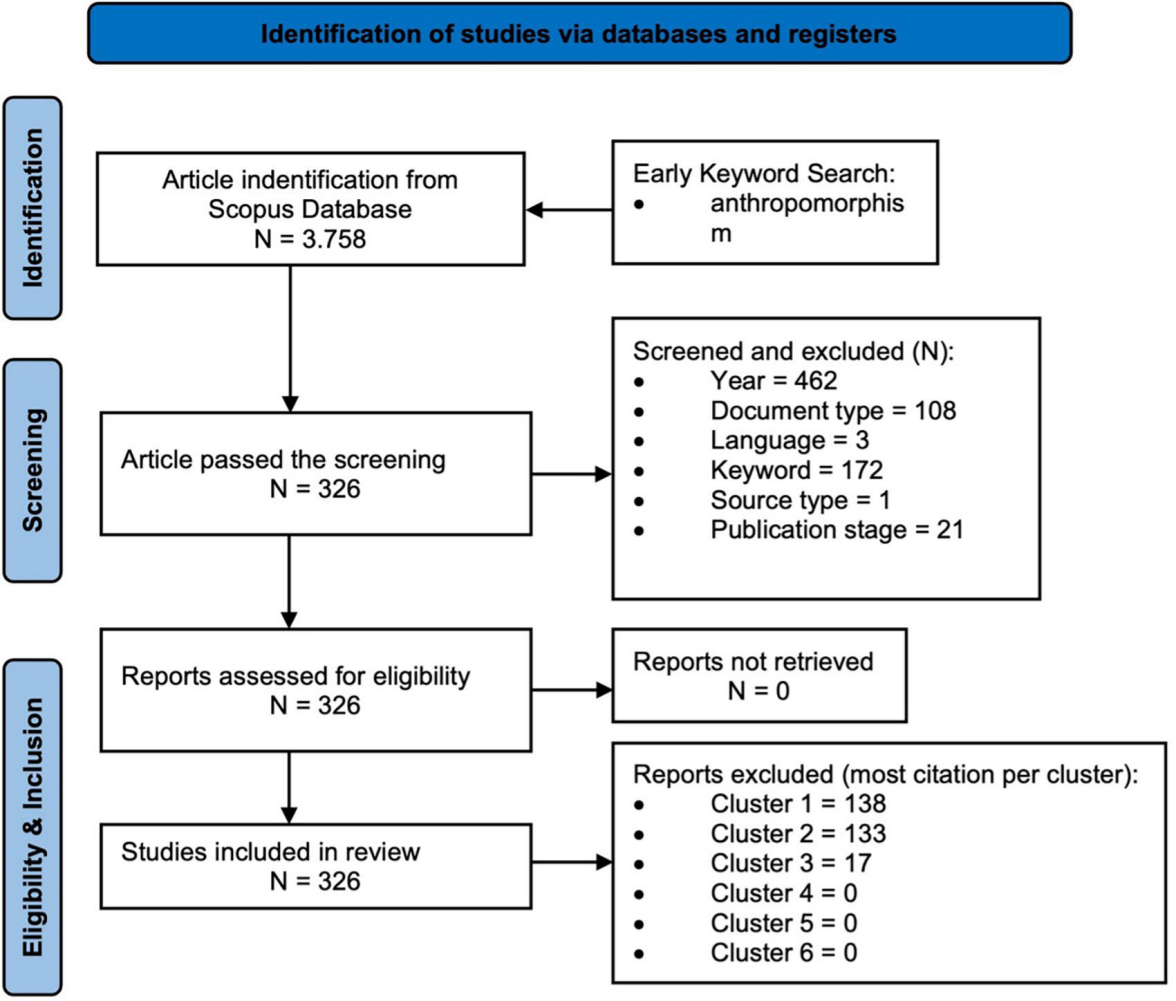
Snapshot of the Research Methodology.

This series of bibliometric methods is used to understand the development of research related to anthropomorphism in the fields of business, management, and accounting. Figure 1 illustrates a flowchart of the bibliometric process, starting with identification, screening, eligibility, inclusion, and knowledge synthesis analysis.

The first step was to identify databases that applied the criteria for identifying Scopus-indexed journal articles. Scopus was chosen because it is an abstract indexing database with full-text links and excellent navigation skills ( Burnham, 2006). Additionally, in the field of social humanities, it possesses unique attributes and represents literature better than the Web of Science ( Venkatesh et al., 2003). Based on the definition of anthropomorphism provided by previous literature ( Payne et al., 2013; Sharma & Rahman, 2022), words related to anthropomorphism, such as “anthropomophism” OR “anthropomorphisms” AND “brand anthropomorphism” AND “perceived anthropomorphism” AND “product anthropomorphism” AND “human like” AND “Marketing” AND “dehumanization” AND “consumer behavior” AND “communication” AND “persuasion”, were used as keywords. The authors then filtered the data and reviewed each relevant article according to the topic of anthropomorphism with several limitations using the formula YEAR-ABS-KEY (“2010-2024”)) AND ( LIMIT-TO (SUBJAREA, “BUSI”)) AND ( LIMIT-TO (DOCTYPE, “ar”)) AND ( LIMIT-TO (PUBSTAGE, “final”)) AND ( LIMIT-TO (SRCTYPE, “j”)) AND (LIMIT-TO (LANGUAGE, “English”)). The results obtained were 326 articles that could be analysed from the period of 2010-2024.

Although anthropomorphism research emerged before 2010 ( Epley et al., 2007; Guthrie, 1995; Vidal et al., 1995), it only began to develop starting in 2010 ( Sharma & Rahman, 2022). Data screening was conducted according to the research objective criteria. Screening was conducted by identifying titles and abstracts. This was done to ensure that the selected articles were appropriate and relevant to each topic. The third step ensured feasibility by thoroughly evaluating articles according to the exclusion criteria. The fourth and final step was inclusion. Inclusion was performed to ensure that all articles met the criteria for the systematic review.

After being deemed suitable, they were extracted for analysis. The analysis was conducted using several tools such as Bibliometric-R (a programming language for statistical computing and graphics) and VOSviewer (a software tool for constructing and visualizing bibliometric networks). Both tools have been frequently used because they allow for relatively easy data analysis and are widely applied in various fields of business research, such as management ( Ellegaard & Wallin, 2015; Zupic & Čater, 2015), accounting ( Setiawan et al., 2023), and marketing ( Donthu et al., 2021).

### 3.2 Screening and determination of results

Database and register identification were carried out by identifying relevant articles in the Scopus database using the keywords “anthropomorphism” or “anthropomorphisms” Then, we used several exclusion criteria (year, subject area, document type, language, keyword, source type, publication stage) to ensure the eligibility of the articles. Additionally, several exclusion criteria were applied to refine the sample and ensure appropriate focus and topics. From 3,758 articles, those that did not match the specified publication year (462 articles) were excluded, as were those outside the field of “business, management, and accounting” (2,665 articles), those that were not articles (108 articles), those written in a language other than English (three articles), those outside the major theme of anthropomorphism (172 articles), those that were not journal articles (one article), and those that had not yet been finalized for publication (21 articles). Ultimately, 326 articles met the inclusion criteria. To ensure that the most relevant studies were considered, article selection was conducted based on the highest number of citations in each cluster (six clusters), and 326 articles were identified for in-depth analysis in the final stage.

### 3.3 Science mapping

Science mapping was used to examine the interactive relationships among the research constituents. Several techniques have been employed in science mapping, including citation, co-citation, bibliographic coupling, co-word, and co-author analyses. The analyses of these techniques were combined to present the bibliometric and intellectual structures in the observed field ( Baker et al., 2021).

**3.3.1 Citation analysis**

Citation analysis was used to identify the most cited publications and authors in the field of anthropomorphism. Citations reflect intellectual connections between publications when one publication cites another ( Donthu et al., 2021). Through citation analysis, we can determine and confirm which research is the most influential and the key figures that shape the understanding of anthropomorphism in the context of business, management, and accounting. Citations are the most objective and straightforward measures of impact in the intellectual realm.

**3.3.2 Co-citation analysis**

Co-citation analysis is conducted to reveal the intellectual structure of a field by examining two publications connected to the reference list of another publication based on similar themes ( Rossetto et al., 2018). The intellectual structure depicted in the co-citation analysis is the discovery of thematic clusters within the study topic. Co-citation analysis is are conducted in bibliometric studies to uncover important publications and the foundational knowledge used ( Kumar et al., 2021).

**3.3.3 Co-word analysis**

In this study, co-word analysis was conducted by examining words that frequently appeared in keywords ( Baker et al., 2021; Kumar et al., 2021). Words that frequently appear in articles have the same thematic relationships. This co-word analysis is used to complement the co-citation analysis to enrich understanding and elucidate the content of the clusters formed in the co-citation analysis. This analysis provides a preview for future research ( X. Chen et al., 2016).

**3.3.4 Co-authorship analysis**

Co-authorship analysis was used here to examine the relationships between authors, institutions, and countries in the context of anthropomorphism in the fields of business, management, and accounting, which are collaborative in nature ( Koseoglu, 2016). This analysis groups and maps based on location to identify the main journals that play a crucial role in sharing research findings. Through co-authorship analysis, novice researchers gain valuable information to collaborate with established researchers in the field of anthropomorphism.

## 4. Finding and discussion

### 4.1 General characteristic of the literature

**4.1.1 Evolution of the number of publications**

**Figure 2.**
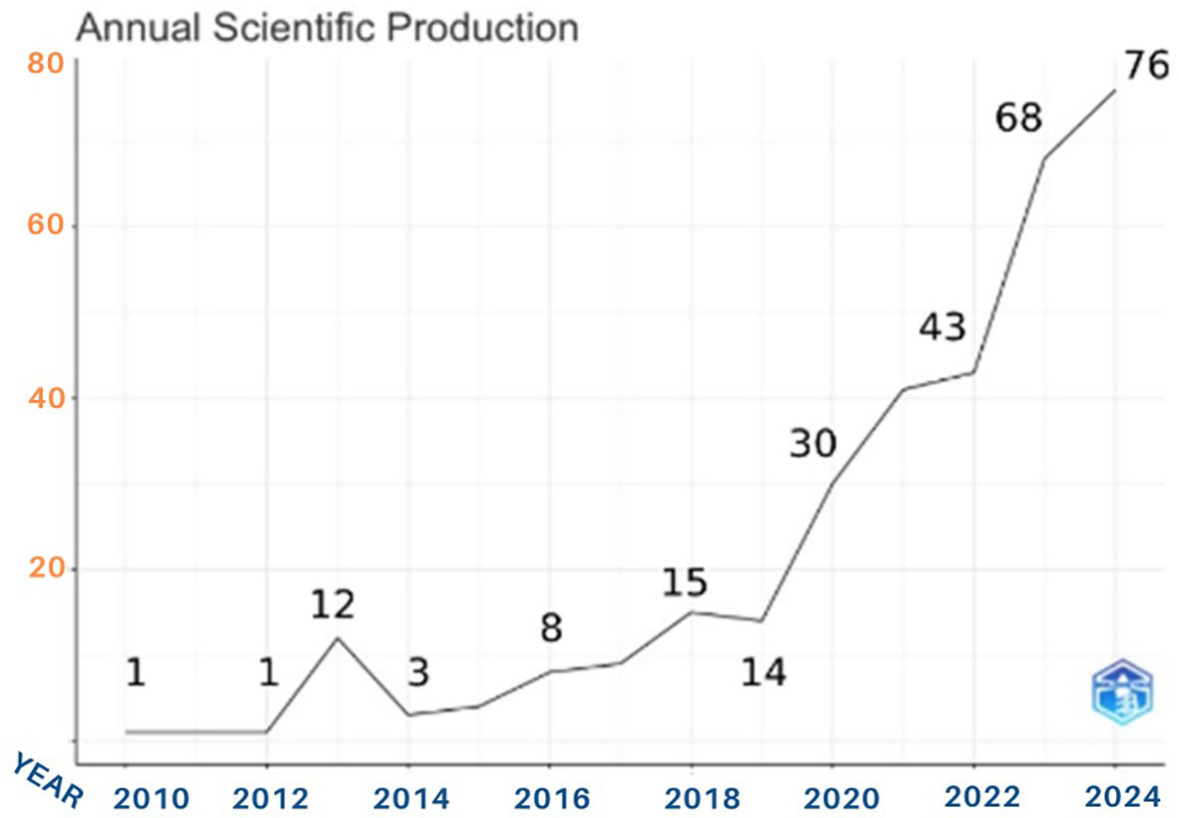
Trends in Anthropomorphism Articles. Source: the authors, made in R-Studio.

The number of anthropomorphism articles produced showed a significant upward trend in terms of the number of publications on anthropomorphism in business, management, and accounting. This can be seen from Figure 2. Over a period of 14 years, an average of 22 published documents were released each year. Based on the general characteristics of the articles, we divided them into four periods: the early years period (2010-2012), gradual increase period (2013-2017), steady growth period (2018-2019), and significant surge period (2020-2024). Only one article was published in the early period of anthropomorphism research. As previously noted, this period is referred to as the early years period (2010-2012). During this 2-year period, the topic of anthropomorphism was not yet familiar to intellectuals; therefore, there were few documents discussing it.

The next period is the gradual increase period. Growth was evident during this period. In 2013, the number of activities with 12 documents published. This indicates an active publication trend, although it declined again in the subsequent years, showing a relatively stagnant phase between 2014 and 2017. During this period, the research was still focused on understanding and mapping the direction of the studies conducted. As there is still no consensus regarding anthropomorphism, this topic has rarely been researched. The years 2018-2019 represent the steady growth period because the stability of publications increases during this period. Scientific support for anthropomorphism research began to appear here. Digital transformations and technology began to emerge in 2019. Advancements in technology allow for interaction with the concept of anthropomorphism, making it increasingly relevant for development. Human-computer interaction has driven an increase in interest in anthropomorphism research.

We refer to the final period from 2020 to 2024 as the significant surge period. In 2020, there was a significant increase in the number of articles published on anthropomorphism (30). This growth became more apparent from 2020 to 2024 with an increase of 153%. The final 4-year period saw a sharp annual increase in the number of publications. The graph shows the highest production rate in 2024, with more than 70 anthropomorphism articles published, marking the peak in scientific activity during the observation period. This is likely due to the global event of the COVID-19 pandemic, which changed all patterns and human activities and encouraged research activities in various fields. Research funding support and government policy changes are needed to sustain life through the development and encouragement of globally relevant research. Post-2020, there has been a shift in research priorities and directions toward fields intersecting with information technology. We conclude that COVID-19 and technological developments have become significant events influencing the increase in the number of publications on anthropomorphism.

**4.1.2 Worldwide distribution network covering various countries and organizations**

**Figure 3.**
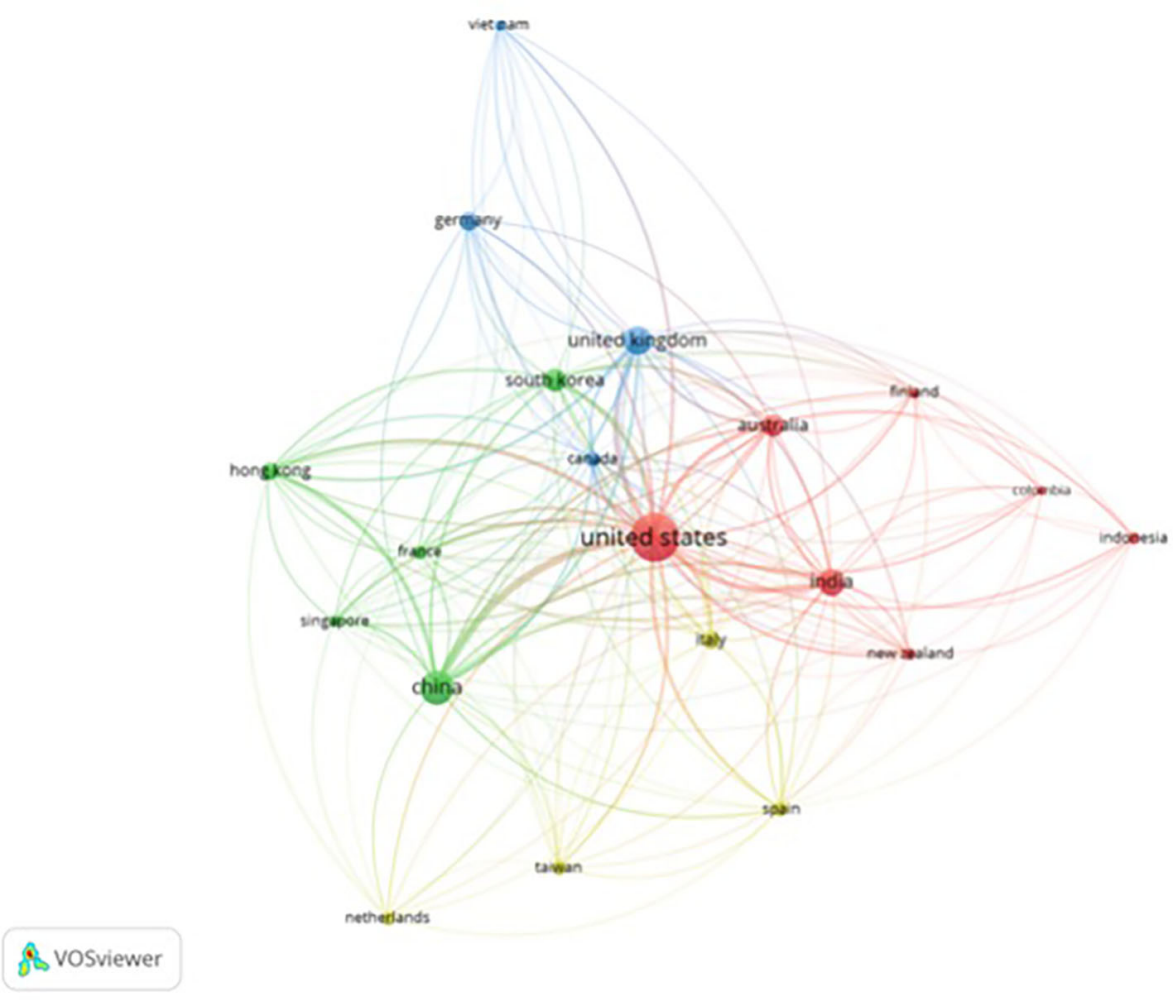
Country Network. Source: the authors, made in VosViewer.

**Figure 4.**
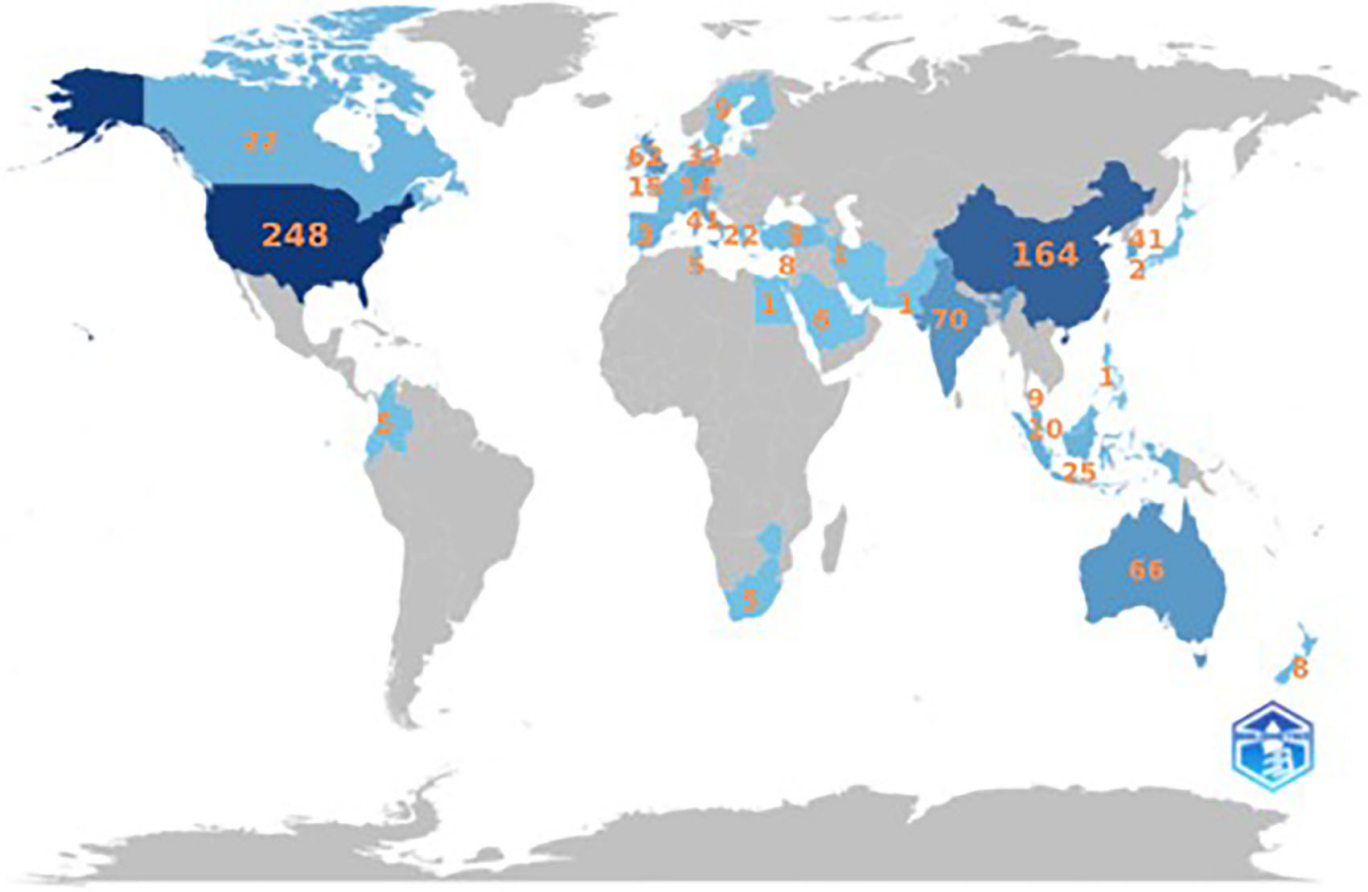
Scientific Production by Country. Source: the authors, made in R-Studio.

It can be seen from Figures 3 and 4 that there are four clusters of countries that have had intellectual connections over the past decade. The United States appears to be the centre of global collaboration and has close ties with China, India, and the United Kingdom. The formation of these four clusters may be due to the similarities in themes and regional areas (geographical proximity) of each country. Cluster 1 included the United States, India, Australia, New Zealand, Finland, Colombia, and Indonesia. This grouping has wide global coverage. Cluster 2 was more focused on technological and economic development; thus, China, South Korea, Singapore, France, and Hong Kong formed this group. Cluster 3 included the UK, Canada, Germany, and Vietnam. Cluster 3 was formed due to the orientation toward fundamental sciences and transatlantic collaboration among these countries. Cluster 4 emphasizes the regional collaboration between Europe and Asia, from Italy, Spain, and the Netherlands to Taiwan. In addition to historical relationships, this grouping reflects a pattern of collaboration with the same research focus.

The United States is the country most involved in the anthropomorphism research citation network with 248 documents, followed by China with 164 documents, India with 70 documents, Australia with 66 documents, and the United Kingdom with 62 documents. Figure 4 illustrates the distribution of anthropomorphism publications worldwide in the business, management, and accounting fields. Of the 43 countries that published on anthropomorphism, four of the 10 countries with the most publications were in Asia, with a total of 300 documents, followed by America with 248 documents, Europe with 136 documents, and Australia with 66 documents. However, the distribution of publications was generally uniform across all regions. This may be because anthropomorphism is used to create emotional connections between individuals and products that apply anywhere ( J. Huang et al., 2024; Wan & Chen, 2021). However, there are differences in perspectives and cultures between countries in the Western and Eastern regions ( Baskentli et al., 2023). Western cultures view anthropomorphism as more cognitive, whereas Eastern cultures tend to view it as related to spirituality.

**Figure 5.**
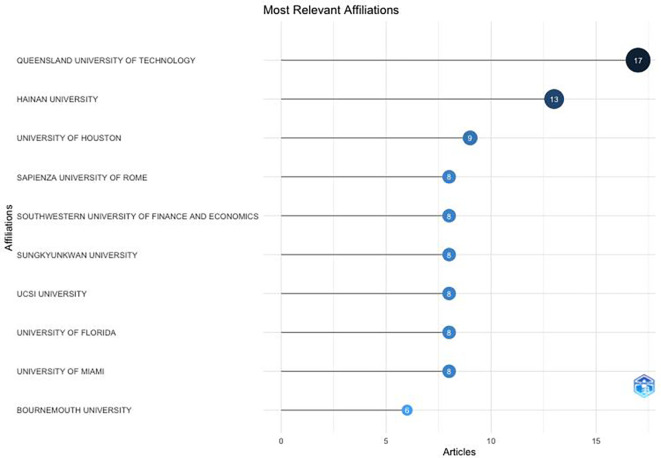
Affiliation Production. Source: the authors, made in R-Studio.

The Queensland University of Technology (Australia) has been the largest contributor to research on anthropomorphism over the past decade ( Figure 5), followed by Hainan University (China) and the University of Houston (United States). Figure 5 also shows that the United States has greater interest in anthropomorphism than other countries. Of the 10 affiliated universities, the majority are from the United States. This also proves that universities have become centres for research and the development of knowledge, particularly that related to anthropomorphism in the fields of business, management, and accounting.

**4.1.3 Insight on relevant and productive journal analysis**

Table 1 presents the top 10 journals that published articles on anthropomorphism, with their reputations indicated by the Scimago Journal Ranking, H-Index, and Cite Score. The Journal of Business Research is the highest-ranked (top-tier) journal with 23 articles related to anthropomorphism. Table 1 also presents the extent of the contributions of these journals to specific research domains, showing robust results and the impact of these scientific journals on the scope of anthropomorphism in the fields of business, management, and accounting research.

**Table 1.** Most Relevant Journal.

No	Name of Journal	Article amount	Cite Score	SJR Rank	H-index
1	Journal of Business Research	23	20.3	Best Quartile (Q1)	265
2	Psychology and Marketing	21	12.1	Best Quartile (Q1)	143
3	Journal of Retailing and Consumer Services	18	20.4	Best Quartile (Q1)	143
4	Technological Forecasting and Social Change	16	21,3	Best Quartile (Q1)	179
5	Journal of Marketing Management	13	8	Best Quartile (Q1)	90
6	International Journal of Hospitality Management	12	21.2	Best Quartile (Q1)	169
7	European Journal of Marketing	11	7.9	Best Quartile (Q1)	154
8	Journal of Consumer Research	11	12.2	Best Quartile (Q1)	220
9	International Journal of Contemporary Hospitality Management	7	11.1	Best Quartile (Q1)	126
10	Journal of the Academy of Marketing Science	7	15.2	Best Quartile (Q1)	207

**Figure 6.**
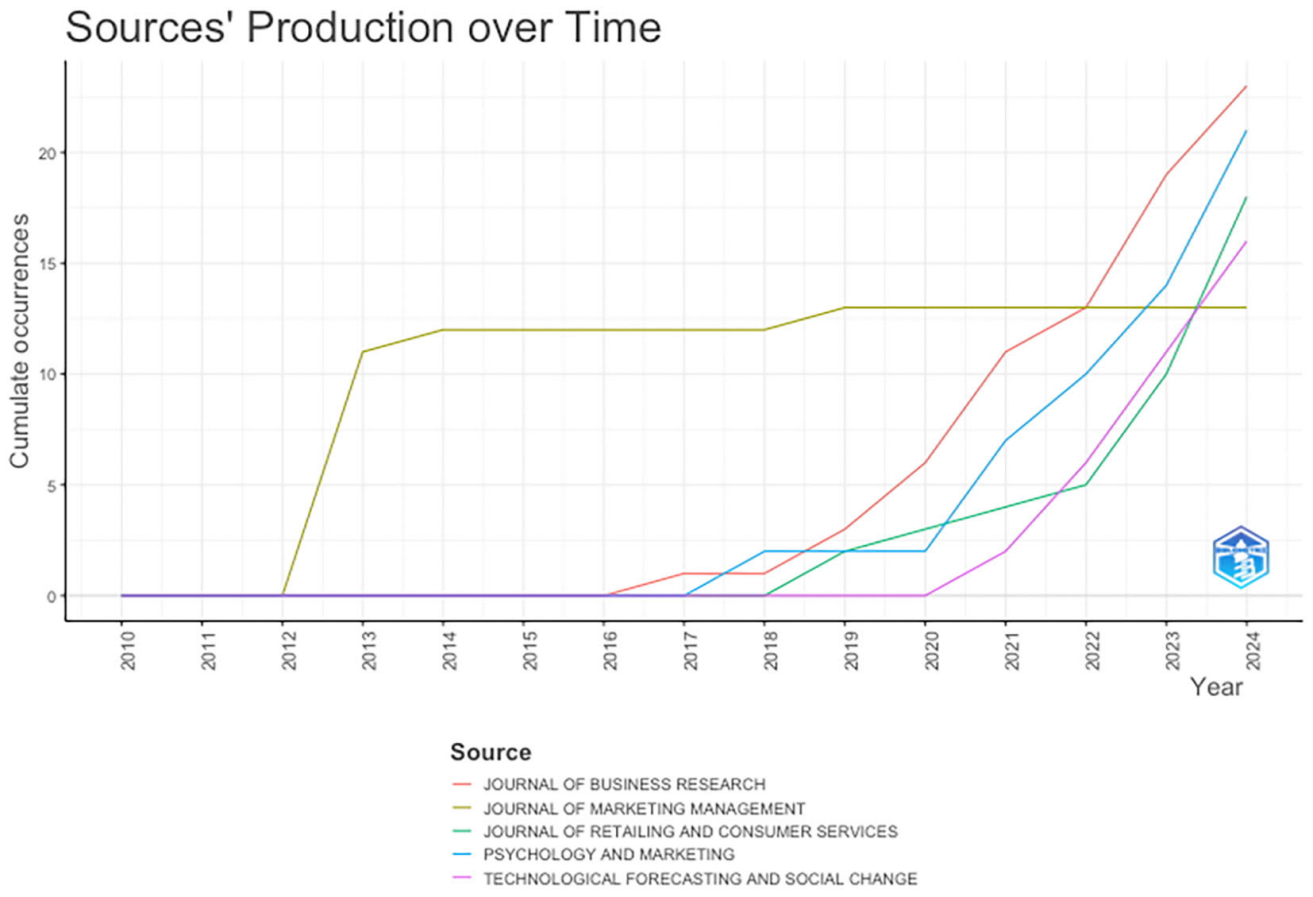
Most Productive Journals. Source: the authors, made in R-Studio.

Figure 6 presents the trend in the number of publications by the TOP five journals that have published on anthropomorphism over the past decade. The Journal of Marketing Management has been a pioneer in publishing anthropomorphism articles since 2013. The Journal of Business Research began publishing anthropomorphism articles in 2017 and continued to increase significantly until 2024, becoming the journal with the most anthropomorphism articles published. The Journal of Retailing and Consumer Services and Psychology and Marketing has also shown the same trend since 2018-2019, while Technological Forecasting and Social Change began publishing anthropomorphism articles in 2020, following the beginning of theCOVID-19 pandemic. This could be due to the pandemic, during which technological innovation developed rapidly and continuously along with increasing academic research support.

Interestingly, among the top five journals, the Journal of Marketing Management is the only one that has consistently published an average of 12 documents per year from 2013 to 2024. We tried to highlight this and map out several factors that might underlie it. 1) The Journal of Marketing Management focuses only on more specific themes, namely marketing management and marketing strategy, compared with the other four journals. In addition, it discusses the fundamental marketing issues that are relevant each year. 2) The Journal of Marketing Management is consistent with marketing research topics and is not overly influenced by the demand for popular new research trends. 3) The authors contributing to the Journal of Marketing Management are established writers who have consistently written and published the same research findings.

### 4.2 Network analysis

**4.2.1 Citation network: The role of the corresponding author in driving impactful publications**

**Figure 7.**
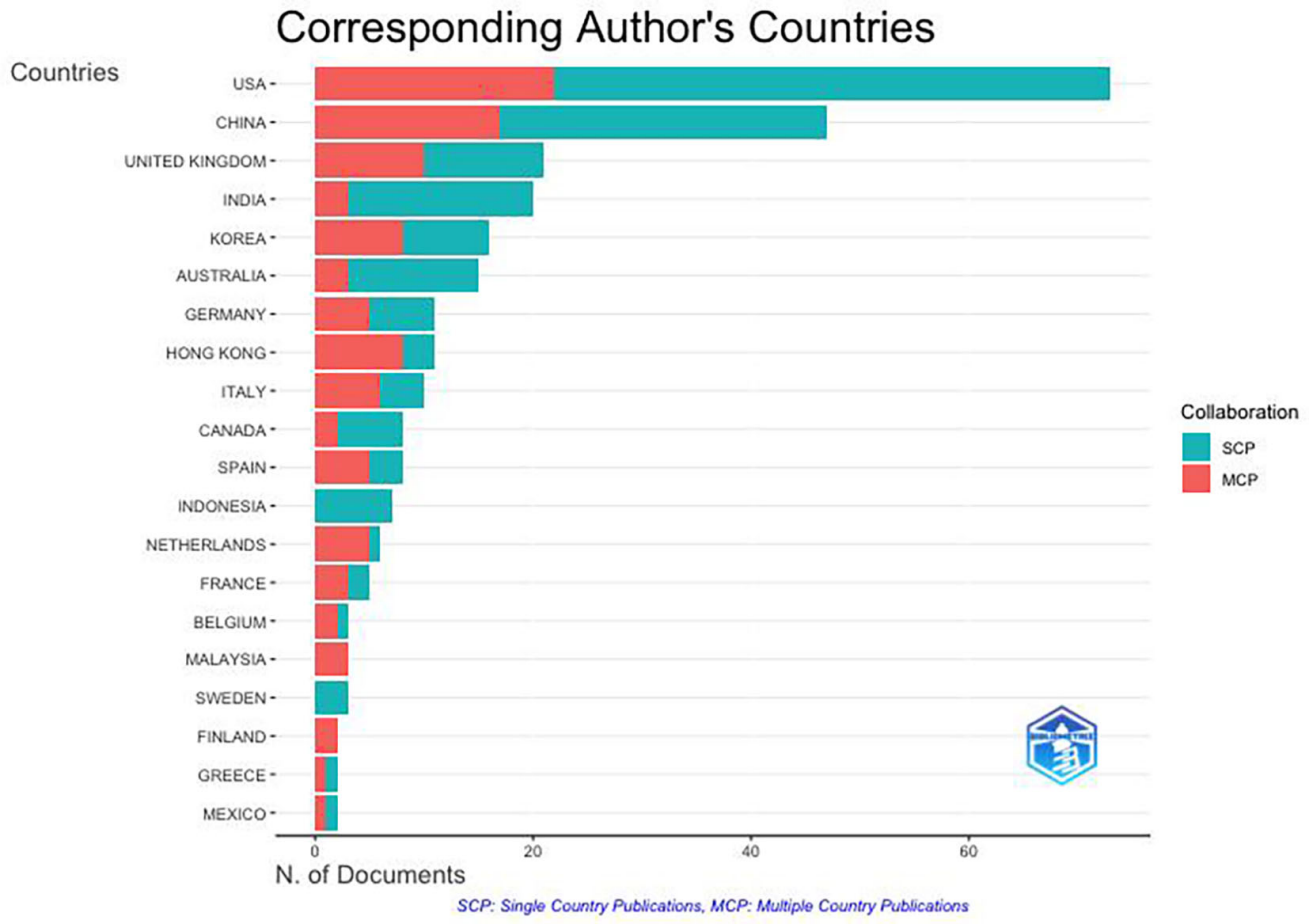
Collaboration Patterns by Corresponding Author’s Country. Source: the authors, made in R-Studio.

The dynamics of anthropomorphism research and collaboration patterns among authors, both within a single country and between countries, were visible ( Figure 7). Most authors have collaborated globally, indicating that there is an awareness of the importance of building international collaborations to expand the impact of intellectual development and deepen knowledge sharing on the same topic. The United States and China are the largest contributors to the development of anthropomorphism in business, management, and accounting. It can be concluded that there is a difference in collaborative styles between Western and Eastern countries. Western countries, such as the United States, England, Germany, France, Belgium, Finland, and Spain, collaborate more with authors from different countries. Eastern countries such as China, India, Korea, and Indonesia collaborate with authors within the same country. Scholars from Eastern countries are expected to strengthen global collaboration and contribute to substantial advancements in anthropomorphism research.

Furthermore, collaboration in anthropomorphism research reflects different thought patterns among authors from developed and developing countries. Authors from developed countries appear to collaborate more with authors from other countries because of their focus on the development and application of technology, as observed in the United States and China. This is unlike writers in developing countries, such as Indonesia and India, who are still exploring anthropomorphism based on unique local cultures within their countries but that are relevant globally. It can be concluded through the authors' collaboration patterns that anthropomorphism has become a relevant topic that intersects not only with technology but also with cultural perspectives that can be strengthened through cross-country collaboration ( Baskentli et al., 2023; Payne et al., 2013). If collaboration on the relevant topics of anthropomorphism, technology, and culture in the fields of business, management, and accounting is pursued, it will enhance the effectiveness and efficiency of business processes in the long term (F. R. Chen et al., 2021).

Table 2 presents the 10 published research articles on anthropomorphism in the fields of business, management, and accounting, along with the authors' names, article titles, publication years, total global citations, and average citations per year. The total global citations refer to the number of citations referenced by other researchers from various disciplines that are accepted in the article database. The average number of citations per year is the average number of articles cited by other researchers from the same or different disciplines.

**Table 2.** The 10 Most Citated Authors.

No.	Author name	Article title	YoP	TGC	TC/Y
1	Mende, M., Scott, M. L., van Doorn, J., Grewal, D., & Shanks, I. (Netherlands)	Service robots rising: How humanoid robots influence service experiences and elicit compensatory consumer responses	2019	666	95
2	Adam, M., Wessel, M., & Benlian, A. (Germany)	AI-based chatbots in customer service and their effects on user compliance	2020	516	103
3	Pillai, R., & Sivathanu, B. (India)	Adoption of AI-based chatbots for hospitality and tourism	2020	460	77
4	Hudson, S., Huang, L., Roth, M. S., & Madden, T. J. (USA)	The influence of social media interactions on consumer–brand relationships: A three-country study of brand perceptions and marketing behaviours	2016	430	43
5	Belanche, D., Casaló, L. V., Flavián, C., & Schepers, J. (Spain, Netherlands)	Service robot implementation: A theoretical framework and research agenda	2019	406	68
6	Sheehan, B., Jin, H. S., & Gottlieb, U. (Australia)	Customer service chatbots: Anthropomorphism and adoption	2020	346	58
7	Van Pinxteren, M. M. E., Wetzels, R. W. H., Rüger, J., Pluymaekers, M., & Wetzels, M. (Netherlands, Finland)	Trust in humanoid robots: Implications for services marketing	2019	317	58
8	Crolic, C., Thomaz, F., Hadi, R., & Stephen, A. T. (UK)	Blame the bot: Anthropomorphism and anger in customer–chatbot interactions	2022	302	76
9	Kim, S. Y., Schmitt, B. H., & Thalmann, N. M. (USA)	Eliza in the uncanny valley: Anthropomorphizing consumer robots increases their perceived warmth but decreases liking	2019	301	43
10	Fournier, S., & Alvarez, C. (USA)	Brands as relationship partners: Warmth, competence, and in-between	2012	279	20

Note: YoP (year of publication); TGC (total global citations); TC/Y (total citations per year)

Based on Figure 7 and Table 2, it can also be concluded that the United States and China are developed countries with centres of technological innovation, such as artificial intelligence, robotics, and the Internet of Things. This explains why anthropomorphism has been widely researched in both countries. It is possible that both countries are developing applications for anthropomorphism research, such as designing more human-like chatbots, virtual assistants, and other functional robots. Moreover, anthropomorphism has become a marketing communication strategy to connect with consumers. Several major e-commerce companies in the United States and China, such as Alibaba and Tesla, have already adopted anthropomorphic strategies to enhance consumer experience through AI mascots and anthropomorphic elements in their autopilot systems. These two examples illustrate the need for further research on anthropomorphism to strengthen the global adoption of anthropomorphism-based technologies. The research competition between the United States and China also shows how each country allocates significant funding investments to the R&D of anthropomorphic technology for business applications.

**4.2.2 Co-citation analysis: Revealing impact through interconnected references**

**Figure 8.**
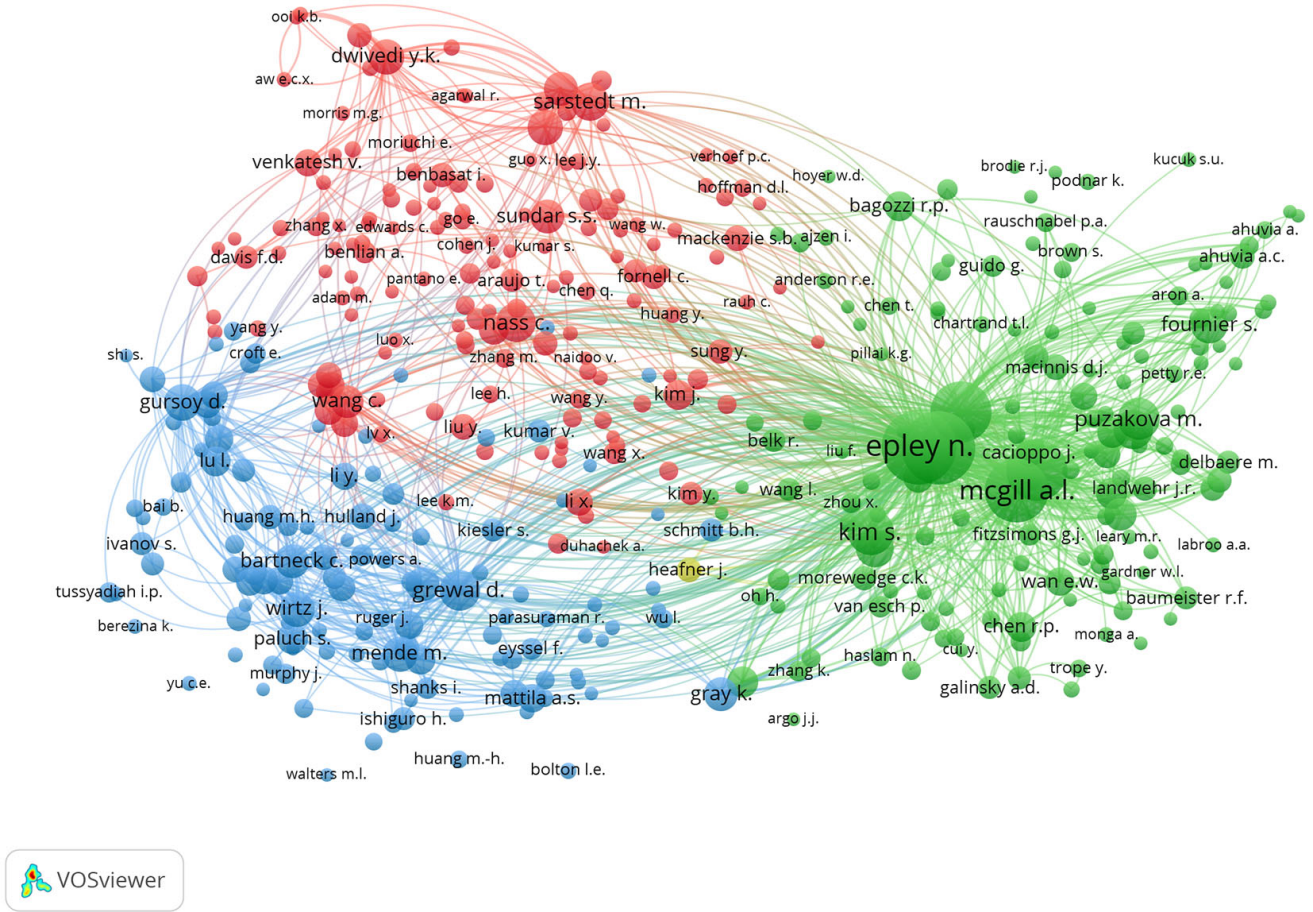
Co-Citation Analysis. Source: the authors, made in VosViewer.

From the visualization in Figure 8, four clusters were identified based on the similarity of topics among the researchers. The green cluster appears to be the largest among the clusters. This indicates that the main themes of psychology and consumer behaviour are interrelated. The largest nodes in this cluster are N. Epley, A. McGill, and S. Kim, indicating that they are most often cited together. The second red cluster involves M. Sarstedt and S. Sundar, who have connectivity in terms of methodology and research framework. The third cluster, coloured blue, focuses more on technological applications with major nodes for X. Wang, D. Gefen, and I. Hu. However, the four clusters are interconnected, meaning that inter-cluster support is needed to build a holistic understanding and create new knowledge that is practically relevant.

**4.2.3. Co-word network analysis: Mapping impact through emerging keywords**

**Figure 9.**
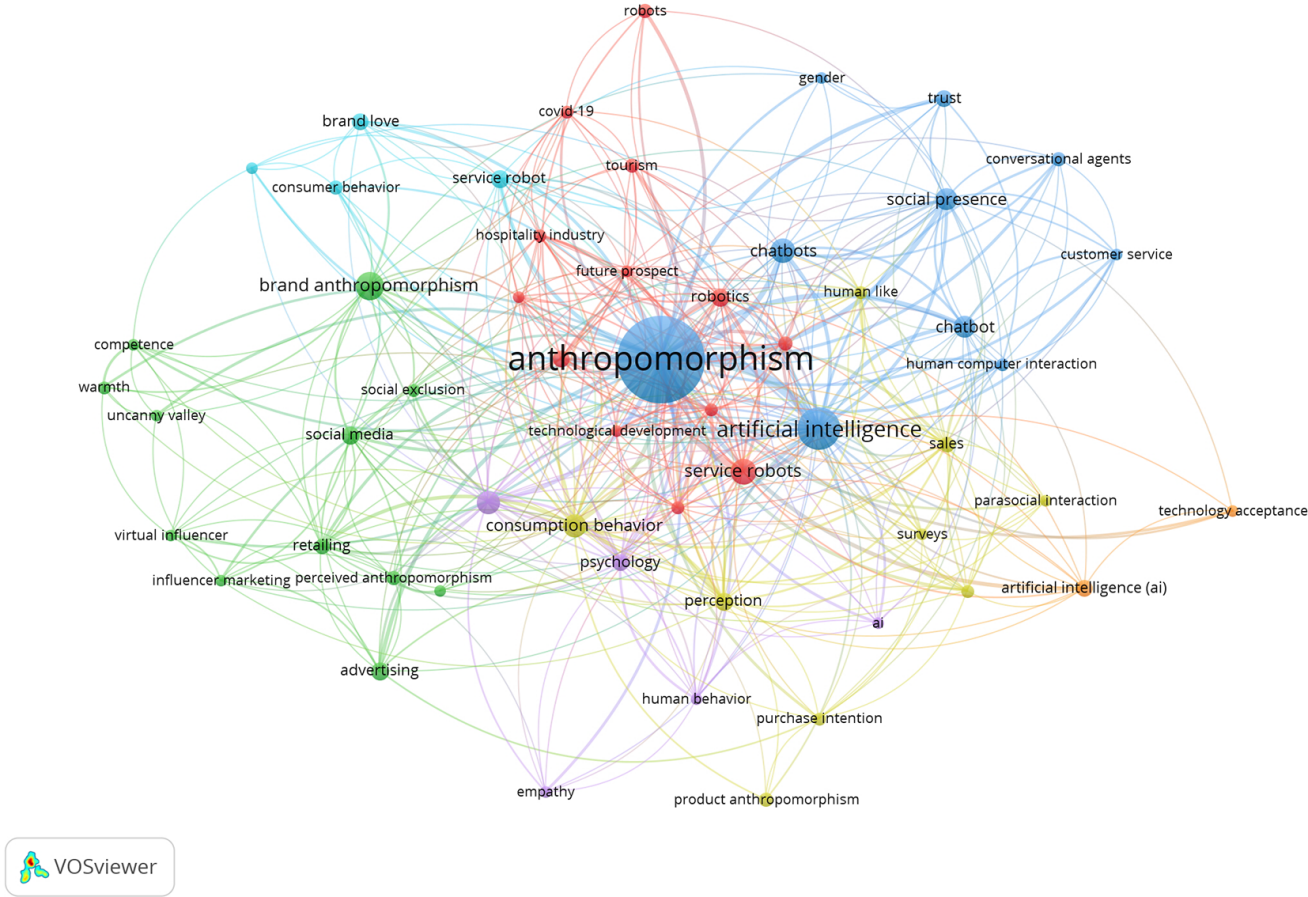
Co-Word Network Anthropomorphism. Source: the authors, made in VosViewer.

The co-word network resulting from the bibliometric analysis visualization using VOS viewer is shown in Figure 9. The displayed visualization includes nodes representing frequently occurring keywords and edges, which are lines connecting nodes based on co-occurrence frequency ( Van Eck & Waltman, 2014). If the node is larger, its frequency of occurrence is higher, and thicker edges indicate a stronger relationship between nodes. Figure 9 highlights the contributions of anthropomorphism to business, management, and accounting. Nodes and edges also grouped several words into the same thematic group and are marked in the same colour. These keywords offer deep insight into their roles and contributions to the evolving needs and challenges of the future business world.

The strongest cluster related to anthropomorphism, marked in navy blue, is technology development. In that cluster, words such as “artificial intelligence,” “human-computer interaction,” and “chatbot” appear. Next, the second cluster in green represents influential communication because words such as “advertising,” “influencer marketing,” and “social media” often appear. The third cluster, in red, with the words “future prospect,” “tourism,” “technology development,” and “hospitality industry,” represents the field of behavioural research. The fourth cluster, in purple, is related to psychological factors. In that cluster, the words “consumption behaviour,” “psychology,” and “empathy” appear. The fifth cluster, coloured yellow with the words “sales,” “perception,” and “purchase intention,” can be concluded to have a theme of consumption behaviour. The sixth cluster, in light blue, with the words "brand love,” “service robot,” and “consumer behaviour,” represents customer satisfaction.

**Figure 10.**
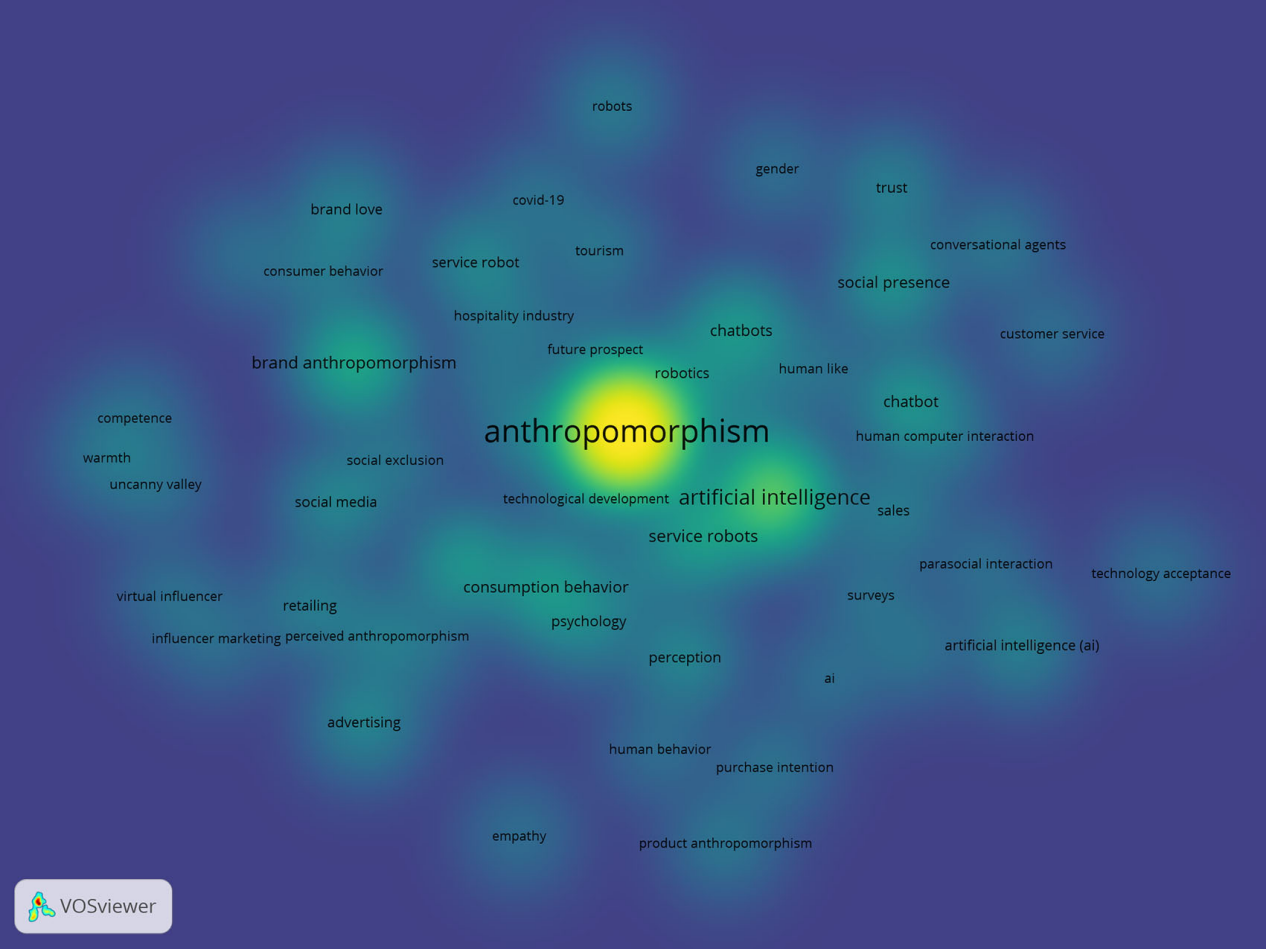
Network Density Anthropomorphism. Source: the authors, made in VosViewer.

The co-word analysis is reinforced by the network density results in Figure 10, which show the strongest collaboration and connectivity on the topic of anthropomorphism in the fields of business, management, and accounting, namely, technology development. The colours strongly associated with anthropomorphism are “artificial intelligence,” “service robots,” and “chatbot.”

**4.2.4 Co-authorship network analysis**

**Figure 11.**
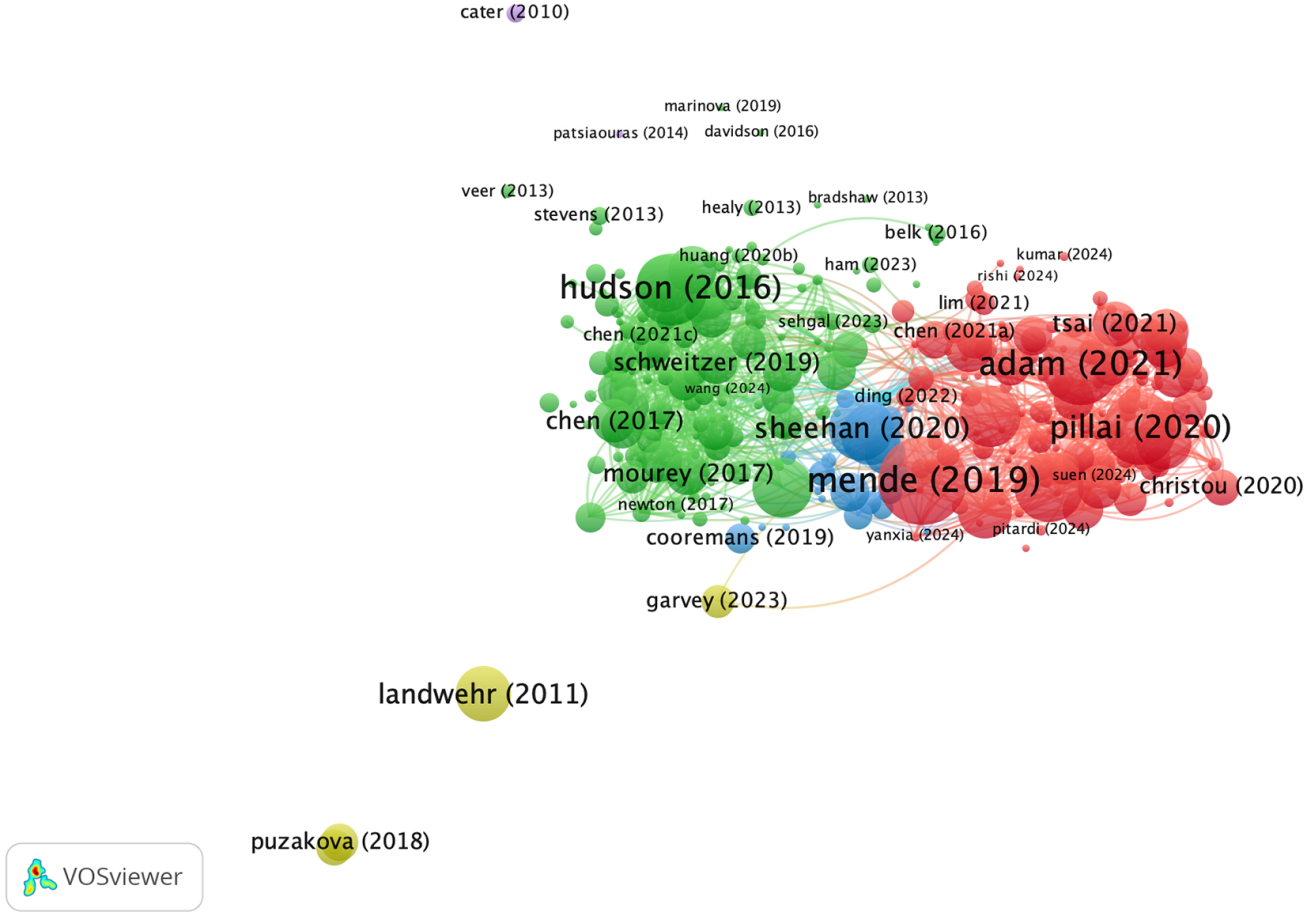
Authorship Network. Source: the authors, made in VosViewer.

Coupling bibliometric analysis includes the grouping of documents based on predetermined criteria, which is a form of data clustering ( Rialti et al., 2019). The strength of document coupling identifies the authors with the widest collaborations and examines how collaboration patterns are formed. Figure 11 illustrates the coupling network generated using VOS viewer, which formed six clusters. Some clusters are interconnected, whereas others are not even though they share the same theme. The red cluster focuses on consumer strategy and digital innovation. It can be seen in this cluster that publications by authors are still very new, and this cluster can be confirmed as having the latest trending theme. The green cluster discusses consumer experiences and satisfaction. The blue cluster specifically examines consumer behaviour and analytical models. The blue cluster is situated between the green and red clusters and acts as an intermediary in the interaction between consumers and technology. The yellow cluster indicates a specific and possibly niche focus as it relates to branding and communication. The purple cluster discusses consumer loyalty, and the orange cluster discusses social innovation in the business context. The authors in each cluster play an important role in building a network of knowledge on anthropomorphism in the fields of business, management, and accounting.

### 4.3 Research clusters on anthropomorphism

The research track record for anthropomorphism from 2010 to 2024 continued to develop, particularly in the fields of business, management, and accounting. Based on a bibliometric analysis, we attempt to summarize specific themes of anthropomorphism the applications of which have implications for consumer interactions, such as psychology and consumer behaviour, communication, brand relationship, consumption behaviour, and technology development.

**4.3.1 AI and service robotics: Advancing human-robot collaboration**

Over the past 4 years, AI and robotic services have been integrated into various industries, changing the manner in which humans interact with technology ( Dwivedi et al., 2019). Humans are beginning to be introduced to digitalization in all fields. The development of AI technology has great potential to support the fields of economics, business, healthcare, industry, and education to enhance efficiency and innovation ( Solntseva, 2018). In the business field, customer assistance and robotic data collection are used to provide personalized consumer experiences. It is important to study the dynamics of human-robot collaboration to ensure an intuitive design that is easy to use and adapt ( Gao et al., 2025; Yang et al., 2022).

Therein lies the importance of research on the design of anthropomorphic robots with human-like features, such as facial expressions, social presence, and communication, which must be relevant ( Mende et al., 2019). The design of anthropomorphic robots must be reliable to enhance relationship closeness and consumer engagement ( Adam et al., 2021; Chung et al., 2023; Sheehan et al., 2020). The goal is for human-like features in AI to provide comfort, build trust, and strengthen emotional connections with users ( Fakhimi et al., 2023). The application of anthropomorphism to robots has been proven to positively influence consumer behaviour, including increasing consumer intentions, purchase decisions, feedback, and technology adoption ( Moussawi et al., 2021). However, it can elicit negative reactions if the service fails or does not meet consumer expectations (Y.-S. Huang & Dootson, 2022). This can be an important consideration for future research, such as privacy and fairness in decision-making, thus requiring the development of algorithms in accordance with the collaborative needs of research fields that combine AI, robotics, psychology, and knowledge ethics ( Belanche et al., 2020).

**4.3.2 Anthropomorphism in branding**

To enhance customer interaction, marketers have begun using anthropomorphism strategies (J. Huang et al., 2024) such as chatbot services, brand mascots, product designs, and personalized advertisements. Chatbots are increasingly designed to resemble human figures, as reflected by their names and conversational styles. Anthropomorphism strengthens the relationship between consumers and brands ( Guido & Peluso, 2015; Y.-T. Huang, 2020; J. Kim et al., 2018). The methods or strategies employed enable a brand to possess human-like qualities, establish emotional connections with consumers, and foster loyalty ( Aaker et al., 2004; Donthu et al., 2021; Fournier, 1998; Hudson et al., 2016).

Strong relational ties influence consumer behaviour (K.-J. Chen & Lin, 2021). Anthropomorphism also affects social control and even the feelings and interactions between humans ( Mourey et al., 2017). Through human-like mascots and relevant product designs, consumers unconsciously experience an improvement in the quality of relationships because they become more attracted to and in love with as well as more understand and competent regarding the brand. The most important aspect is the manner in which anthropomorphic features enhance consumer expectations and provide different experiences for each brand. Through anthropomorphism, customers gain behavioural, cognitive, affective, and social experiences that influence their purchase intentions ( Wang et al., 2022). These experiences have a positive impact on consumer behaviour ( Trung, 2023; Wongkitrungrueng et al., 2020). Anthropomorphism can also raise consumer expectations, which can cause dissatisfaction or distrust if anthropomorphism does not meet consumer expectations ( Barari et al., 2020; S. Kim et al., 2016). Anthropomorphism in branding poses a significant challenge because marketers cannot predict consumer reactions to anthropomorphized brands ( Wan, 2021). The hope is that research on brand anthropomorphism can enhance consumer experience rather than damage the relationship between the brand and consumers. Most importantly, anthropomorphism has become a branding strategy for products, services, and advertisements ( T.-L. Huang & Liu, 2021).

**4.3.3. Anthropomorphic AI interactions**

Anthropomorphism, with its humanlike characteristics, plays an important role in maintaining relationships and interactions with consumers. Anthropomorphism fosters emotional attachment and strengthens the relationship between consumers and brands ( Yang et al., 2022). By exercising a positive influence on consumers, anthropomorphized brands are considered trustworthy and strengthen consumer-brand relationship engagement ( Baskentli et al., 2023; T. Kim et al., 2020). Emotional involvement is reflected in the emergence of a sense of empathy toward the brand ( Lim et al., 2022). Anthropomorphism has an effective impact on brand communication, which, in turn, affects consumer purchase intentions (F. R. Chen et al., 2021; Shao et al., 2020).

Commercial friendships have been established to build empathy with consumers, with service providers as the key to social bonding ( Letheren et al., 2021). Bonding in commercial friendships is built on service evaluations ( Jayanti & Whipple, 2008), service relationships and commitment ( Choi et al., 2021), and behavioural loyalty ( Abosag et al., 2017). The accumulation of anthropomorphism enhances emotional relationships and shapes consumer behaviour, thereby increasing brand loyalty and preferences. Although effective, if not carefully considered, anthropomorphic design can create unrealistic consumer expectations and psychological anxiety related to manipulation ( K. Kim et al., 2019).

**4.3.4 Anthropomorphism and consumer perception**

Anthropomorphism, the attribution of human characteristics to nonhuman entities, is a common marketing strategy used to enhance consumer engagement and brand relationships. Research has shown that anthropomorphized products or brands can enhance consumer preferences and engagement, evoking feelings of familiarity, warmth, and emotional closeness ( Epley et al., 2007). Products with a human-like appearance or characteristics tend to be attractive and boost sales ( Aggarwal & McGill, 2012; Puzakova et al., 2013). Consumer perspectives can change owing to anthropomorphism, such as sidelining price, function, and objective attributes in favour of subjective and emotional considerations ( H. M. Kim & Kramer, 2006).

For certain products, consumers prefer human agents because they require sincerity ( Waytz et al., 2010). In conclusion, the use of anthropomorphism in products or brands must be carefully adjusted according to the product context and the target market. The use of anthropomorphic strategies that are excessively aligned or not aligned with the target market can lead to negative perceptions, especially if human features do not align with the product's function or purpose. If applied effectively, it not only enhances consumer perception but also fosters a stronger emotional bond between the product brand and consumers, leading to increasingly competitive satisfaction.

**4.3.5 Anthropomorphism in tourism**

Research on anthropomorphism is becoming increasingly important for tourism development. The connection between human-like qualities in objects, animals, and visiting experiences is used to enhance the appeal and engagement of tourist destinations. Anthropomorphic design creates an image and impression of comfort that is relevant to a consumer's cultural context ( Landwehr et al., 2011). Cultural attractions at tourist destinations are based on human characteristics, thereby building emotional connections with visitors. Visitor involvement shapes the positive perception of a tourist destination as friendly, comfortable, and memorable ( Wan et al., 2016).

The effectiveness of anthropomorphism in tourism, such as stories of marine animals depicted as "friends" with friendly personalities, makes visitors feel that their interactions are more meaningful ( Cater, 2010; F. Chen et al., 2023). This story has implications for empathy and positive attitudes toward conservation efforts. For tourism operators, the use of anthropomorphism not only provides visitors with an experience and increases satisfaction but also offers significant long-term benefits. The presence of satisfaction and repeated visits creates a lasting impression, builds emotional connections, and promotes positive word-of-mouth promotion. When consumer preferences increasingly prioritize experiences, anthropomorphism offers a distinction in building deeper relationships.

**4.3.6 Chatbots in commerce**

The widespread use of AI-based chatbots is changing how business and trade interactions occur. Chatbots utilize algorithms and language processing to simulate human conversations with non-human entities and make them appear human-like. Research suggests that chatbots play an important role in shaping consumers’ perceptions of products and services. How can chatbots guide consumers in providing information related to product/service features, alternative choices, and decision-making? Chatbots provide a more engaging shopping experience and build trust ( Y.-S. Huang & Dootson, 2022). Chatbots can replace humans in the consumer shopping experience. Communication delivered by a chatbot, both in language and speaking style, resembles that of a human, which fosters a sense of social presence that strengthens relationships with consumers. When consumers feel that the chatbot is competent, they follow its recommendations ( Martin et al., 2020). However, becoming dependent on chatbots is not beneficial for consumers. In business, it is important to maintain a balance between machine automation and genuine human services so that chatbot services do not hinder the business. As technology advances, research on chatbots, emotional intelligence, and contextual understanding of anthropomorphism becomes important to optimize business performance.

### 4.4 Promising research topics

**Figure 12.**
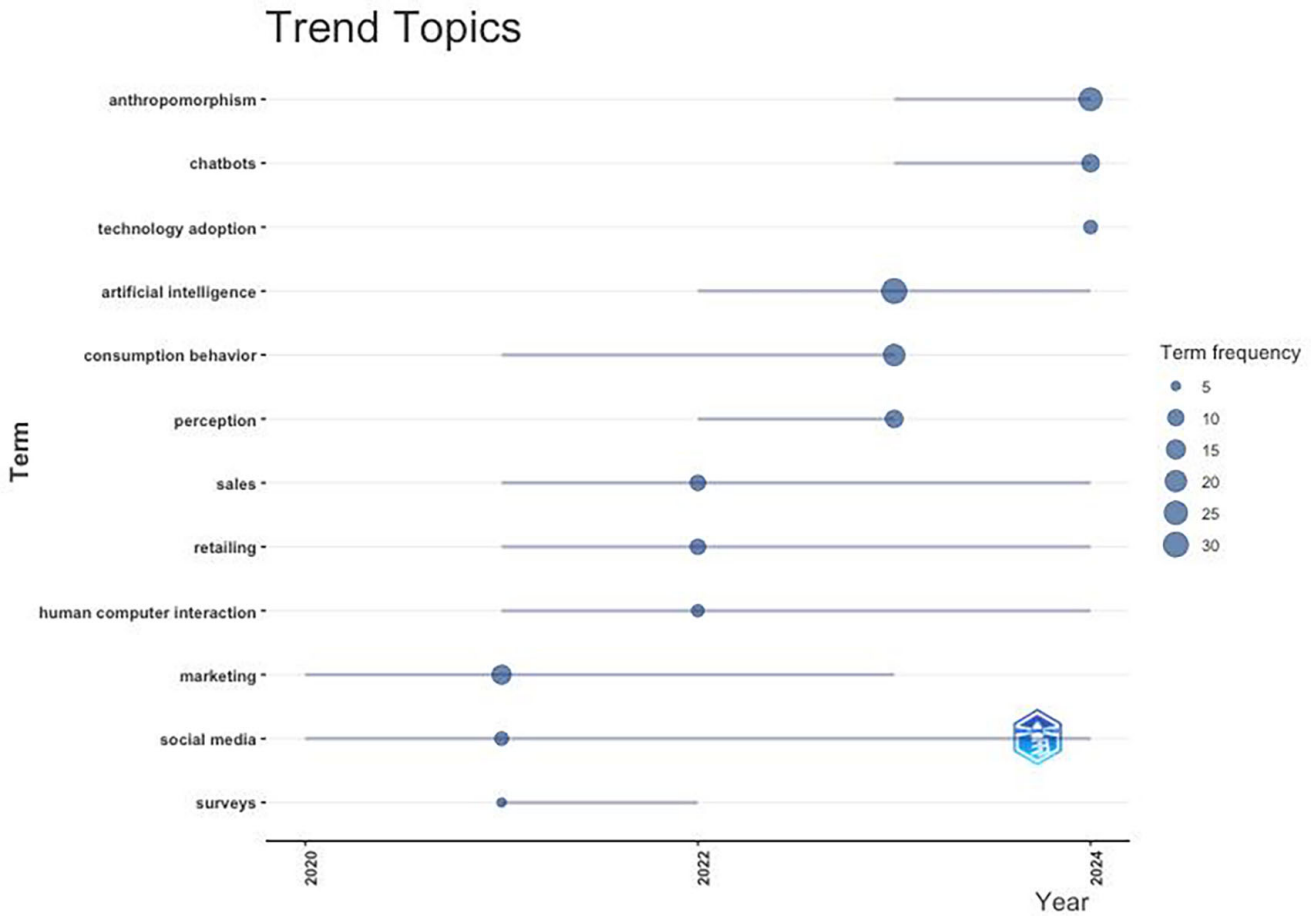
Trending Topics. Source: the authors, made in R-Studio.

Figure 12 Bibliometric-R analysis identifies promising future topic trends that are beneficial for both academia and industry. This research topic trend can provide opportunities for real and beneficial contributions to technological innovation and human interaction.

- **Consumer Behaviour.** How anthropomorphism affects trust, consumer engagement, and brand loyalty; exploring the limits of anthropomorphism to avoid the “uncanny valley.”
- **Artificial Intelligence and Chatbots**. How anthropomorphism in AI can be used to create a more personal, inclusive, and efficient consumer experience, exploring its impact on consumer decision making.
- **Technology Adoption**. Exploration of factors influencing technology adoption through anthropomorphism, such as risk perception, usefulness, and trust; how the patterns of technology adoption vary across cultures and regions.
- **Human-Computer Interaction**. How to create interaction designs between humans and technology and how the exploration of anthropomorphic designs can enhance the effectiveness and comfort of consumers in using AI devices, robotics, and other technological applications.

## 5. Conclusion, Implications, Contributions, and Limitations

This study highlights the increasing interest in scientific publications on anthropomorphism in the fields of business, management, and accounting to address several questions regarding the evolution of anthropomorphism research and consistent investigation of the topic's development since 2010, reaching 326 documents in 2024. The increase in research documents over these 14 years is due to several factors. The first is the shift in research priorities and direction toward fields related to information technology post-COVID-19 pandemic. Second, the active involvement of countries such as the United States, China, India, and Australia as well as university-level research organizations in collaboration plays an important role in the development of anthropomorphism. Third, an increasing number of reputable international journals are accepting anthropomorphic publications. Ultimately, the momentum of the changing trend in the development of anthropomorphism, led by N. Epley, A. McGill, and S. Kim, who focused on psychology and consumer behaviour, shifted toward information technology after 2020. The major challenge in the study of anthropomorphism today is how to avoid the "uncanny valley" because technology cannot fully replace the role of humans in social interactions. Although these challenges are very difficult to overcome, the topic of anthropomorphism offers potential for further research.

This study plays an important role and contributes significantly to the development of business and daily life. Anthropomorphism is a key element in the integration and adoption of increasingly complex technologies. Moreover, anthropomorphism has the potential to transform the way businesses operate as well as customer service and competition in the global market. With the continuous development of technology, anthropomorphism is projected to become increasingly relevant in creating personal experiences, loyalty, and adaptive business ecosystems.

Despite its important contributions, this study still has limitations. First, the analysis only covers the period beginning when the topic began developing, not when it first emerged (before 2010). Subsequent research could identify the emergence of anthropomorphism from the beginning to obtain more comprehensive results. Second, the analysis was exclusively limited to articles published in English in Scopus-indexed journals, and excluded articles written in Spanish, French, or Chinese. Combining articles in different languages that authors do not understand can lead to errors in the interpretation process of a bibliometric analysis. Future research should analyse documents using a larger number of database sources to obtain results with a broader scope. Third, the bibliometric analysis only analysed the dataset of keywords, titles, abstracts, citations, and affiliations. Future research should conduct systematic literature reviews using comprehensive data. Finally, this study only used the analysis results from Bibliometric-R and VOS viewer. Future studies could integrate other visualization analysis tools to obtain more comprehensive results.

## Ethics and consent

Ethical approval and consent were not required

## Statement using AI tool

During the preparation of this work the authors used Chat-GPT in order to paraphrase. After using this tool/service, the authors reviewed and edited the content as needed and takes full responsibility for the content of the publication.

## Data Availability

No data associated with this article. Reporting guidelines: Zenodo: Dataset: Anthropomorphism Unveiled: A Decade of Systematic Insights in Business and Technology Trends [Data set]. Zenodo. https://doi.org/10.5281/zenodo.14908932. ( Pramesti, D. A., et al., 2025). The project contains the following extended data:
1.
PRISMA flow diagram for Anthropomorphism Unveiled.pdf
2.
Dataset of articles retrieved from Scopus Database.csv
3.

PRISMA_Checklist_Literature Data Integration of Anthropomorphism.docx Data are available under the terms of the Creative Commons Attribution 4.0 International license (CC-BY 4.0) ( https://creativecommons.org/licenses/by/4.0/).
