# Supplementary material for: Anthropomorphism Unveiled: A Decade of Systematic Insights in Business and Technology Trends
Source: F1000Res. 2025 Sep 4;14:281. Originally published 2025 Mar 10. [Version 2] doi: 10.12688/f1000research.162157.2 (PMC12547380; doi:10.12688/f1000research.162157.2)
Supplement: Peer review history

# Reviewer report: Reviewer response for version 2

**DOI:** 10.5256/f1000research.186512.r442080

**Published:** 2026-01-06

**Authors:** Raghava R. Gundala

**Affiliations:** 1 University of Wisconsin-Parkside, Kenosha, USA

This manuscript presents a systematic literature review and bibliometric analysis of anthropomorphism in business, management, and accounting from 2010 to 2024. The authors have effectively used PRISMA protocols alongside bibliometric tools (Bibliometrix-R and VOSviewer) to map the field's evolution. The revised version demonstrates significant improvement in theoretical depth, particularly with the integration of psychological and socio-cultural paradigms to explain the divergence between Western and Eastern perspectives. The identification of six distinct research clusters provides a valuable roadmap for future scholars.

 Below is my assessment, based on the specific review guidelines:

 1. Is the work clearly and accurately presented, and does it cite the current literature?

 Yes.

 The manuscript is well-structured and written. The authors have adequately defined the study's scope (2010–2024), and the timeline is sound, capturing the pre- and post-COVID-19 evolution of the topic. The literature cited is current and relevant, effectively covering foundational theories (e.g., Epley et al.) as well as emerging trends in AI and robotics up to 2024. The integration of recent studies regarding the "uncanny valley" and cross-cultural consumer behavior enhances the accuracy of the thematic discussion.

 2. Is the study design appropriate, and does the work have academic merit?

 Yes.

 The study design, which combines a Systematic Literature Review (SLR) with bibliometric mapping, is well-suited to synthesizing a fragmented field such as anthropomorphism. Academic merit is evident in the study's ability not only to count citations but also to reveal intellectual structures and collaboration networks. The shift in focus identified by the authors—from purely psychological consumer behavior to technology-driven applications (AI, chatbots)—is a significant contribution that validates the merit of this longitudinal analysis.

 3. Are sufficient details of methods and analysis provided to allow replication by others?

 Yes.

 The authors have improved the methodological transparency in this version.

  

- **Protocol:** The inclusion of the PRISMA flow diagram (Figure 1) within the main text outlines the identification, screening, and inclusion steps.
- **Search Strategy:** The specific search string (keywords and Boolean operators) and exclusion criteria (e.g., subject area "BUSI," language "English") are explicitly stated, which supports replicability.
- **Tools:** The use of specific software (R-Studio and VOSviewer) is documented.

4. If applicable, is the statistical analysis and its interpretation appropriate?

 Yes.

 The bibliometric indicators (co-citation, co-word, and co-authorship analysis) are correctly applied. The interpretation of the resulting maps is insightful. Specifically, the authors' interpretation of the country clusters (Figure 3) to discuss the "Western vs. Eastern" collaborative styles and cultural paradigms adds analytical depth, often missing in standard descriptive bibliometrics. Figures 9 and 10 show network density and co-occurrence data that strongly support the identification of the six thematic clusters.

 5. Are all the source data underlying the results available to ensure full reproducibility?

 Yes, with a minor correction needed.

 The authors have laudably provided an "Extended data" section with a Zenodo link containing the dataset and PRISMA checklist. However, there is a contradictory statement in the text under "Underlying data," which states, "No data associated with this article," immediately followed by the Zenodo link.

**Recommendation:** The authors should remove the phrase "No data associated with this article" to avoid confusion, as they have provided the dataset.

 6. Are the conclusions drawn adequately supported by the results?

 Yes.

 The bibliometric findings demonstrate strong alignment with the conclusions. The projection of "Promising Research Topics" (Figure 12)—such as AI, technology adoption, and human-computer interaction—is directly supported by the "Trend Topics" analysis, which shows the recency of these keywords. The study's conclusion effectively depicts the progression of anthropomorphism from a branding strategy to a crucial element in technological interfaces. The authors also appropriately acknowledge the limitations of relying exclusively on the Scopus database and English-language articles.

**Final Recommendation:**

 The article is scientifically sound, methodologically robust, and offers clear contributions to the field. I recommend acceptance, subject to the minor correction regarding the data availability statement mentioned above.

Are the rationale for, and objectives of, the Systematic Review clearly stated?

Yes

Is the statistical analysis and its interpretation appropriate?

Yes

If this is a Living Systematic Review, is the ‘living’ method appropriate and is the search schedule clearly defined and justified? (‘Living Systematic Review’ or a variation of this term should be included in the title.)

Yes

Are sufficient details of the methods and analysis provided to allow replication by others?

Yes

Are the conclusions drawn adequately supported by the results presented in the review?

Yes

Reviewer Expertise:

Marketing

I confirm that I have read this submission and believe that I have an appropriate level of expertise to confirm that it is of an acceptable scientific standard.

# Reviewer report: Reviewer response for version 2

**DOI:** 10.5256/f1000research.186512.r417303

**Published:** 2025-10-22

**Authors:** Mahir Pradana

**Affiliations:** 1 Telkom University, Bandung, West Java, Indonesia

Glad to review this article. I see that as a review paper, it addresses clear research gap, namely the lack of integrative mapping studies in these fields, and employs established bibliometric tools and protocols (Bibliometrix-R, VOSviewer, PRISMA). I personally believe the topic is timely given the increasing relevance of human-like AI and virtual agents in marketing and management.

 I also see that the analysis recognizes anthropomorphism’s “critical function” in marketing communication, a relevant and underexplored linkage. Some minor points: the abstract assumes reader familiarity with anthropomorphism’s role in business contexts, but I believe the reader would benefit from the definition and exemplification of it it (e.g., anthropomorphic brands, AI chatbots, mascots).

 Some findings are too general (at least that how I feel); the focus in concrete results needs to be emphasized (e.g., dominant theories, key countries, or emerging subthemes).

 The authors also should highlight the value-added contributions (how this synthesis advances understanding or future agendas) should be emphasized at the end.

 The literature review itself feels redundant for me, because the whole article IS a review. You can focus on already published literature reviews about the similar topics. To  strengthen the depth of the article, the discussion could benefit from theoretical anchoring, e.g., linking again to frameworks like the Technology Acceptance Model (TAM), Social Presence Theory, or the Computers Are Social Actors (CASA) paradigm. Then drawing line between antecedents and consequences of anthropomorphism in these contexts (e.g., what drives anthropomorphic design vs. what outcomes it produces).

 The discussions section must reveal original contributions rather than describing previous work in results and onwards. Focus on prioritizing what matters the most and categorizing the results in a novel way. You also need to explain what new knowledge this manuscript adds to the literature by using citations in the discussions section.

 The identification of six clusters — *AI and service robotics, branding, anthropomorphic AI interactions, consumer perception, tourism, and chatbots in commerce* — provides a clear taxonomy that captures the diversity of research foci. The explanations for each cluster are concise yet meaningful, balancing empirical examples and conceptual interpretation, please just make sure to show the alignment with previous literatures or what novel contributions you draw.

 To me, the author goes beyond merely summarizing publication trends. The section successfully connects macroeconomic aspect (growth due to COVID-19, global collaboration) to microlevel insights (six research clusters). This dual perspective shows methodological breakthrough and well-analyzed definitions.

 Some paragraphs are too long, making them slightly challenging to read continuously. Some sentences — especially in the long cluster discussion — could be split or reorganized to improve rhythm and reader accessibility.

 Example:

 “...but require careful calibration to mitigate the discomfort associated with the phenomenon described in the Branding section...” can be reorganized shorter but more compact.

 For the conclusion, I suggest the authors to categories the implications section rather than presenting them in one big sections. Divide types of implications (theoretical or practical), then limitations and future contributions. Please do make sure to answer the research questions you offered in the beginning.

Are the rationale for, and objectives of, the Systematic Review clearly stated?

Yes

Is the statistical analysis and its interpretation appropriate?

Partly

If this is a Living Systematic Review, is the ‘living’ method appropriate and is the search schedule clearly defined and justified? (‘Living Systematic Review’ or a variation of this term should be included in the title.)

Not applicable

Are sufficient details of the methods and analysis provided to allow replication by others?

Yes

Are the conclusions drawn adequately supported by the results presented in the review?

Yes

Reviewer Expertise:

business and marketing

I confirm that I have read this submission and believe that I have an appropriate level of expertise to confirm that it is of an acceptable scientific standard.

# Reviewer report: Reviewer response for version 2

**DOI:** 10.5256/f1000research.186512.r411741

**Published:** 2025-09-11

**Authors:** Taeshik Gong

**Affiliations:** 1 Hanyang University, Ansan, South Korea

The revised manuscript represents a substantial improvement over the original version. The authors have carefully addressed earlier concerns regarding clarity, methodological transparency, and theoretical integration. Overall, this is a timely and ambitious systematic review that synthesizes anthropomorphism research in business, management, and accounting. The paper has value for both academics and practitioners interested in understanding the evolution of this domain and identifying future research directions. At the same time, several issues remain that require further refinement before the manuscript reaches publishable quality.

**1. Strengths**

 The revision demonstrates a clearer narrative and improved coherence, particularly in the introduction and conclusion. The integration of psychological and sociocultural paradigms provides a more compelling theoretical foundation than in the earlier version. Methodologically, the paper now includes greater transparency by detailing the PRISMA process, inter-coder reliability, and data cleaning procedures. The bibliometric mapping across six thematic clusters is systematically presented and offers readers a structured overview of anthropomorphism research between 2010 and 2024. Importantly, the manuscript now provides more nuanced cross-cultural comparisons (e.g., Western vs. Eastern perspectives) and explicitly identifies gaps in the literature, strengthening its scholarly contribution.

**2. Areas for Improvement**

 The theoretical framing should be further strengthened by explicitly linking the six identified thematic clusters to the two overarching paradigms of psychological and sociocultural perspectives. At present, the paradigms are introduced conceptually but are not consistently applied to interpret the bibliometric results. Making this connection more explicit would sharpen the paper’s theoretical contribution and show how the bibliometric mapping advances theory.

 The methodological transparency has improved, but the PRISMA flow diagram should be presented in the main manuscript rather than only in extended data. This would make the inclusion and exclusion process clearer to readers. In addition, definitions of bibliometric techniques such as co-citation, coupling, and co-word density should be explained in plain terms for an interdisciplinary audience. It would also be helpful to briefly justify the exclusive reliance on Scopus and reflect on how the choice of database shapes the findings.

 The presentation of results remains overly descriptive. Long passages on publication counts, country networks, and journal rankings could be shortened and synthesized. Greater emphasis should be placed on drawing out the theoretical and managerial implications of these patterns rather than reporting statistics in detail. Concise tables or figures could replace lengthy textual descriptions.

 The discussion section would benefit from a deeper interpretive layer. The manuscript now touches on cultural comparisons, but the implications for theory, practice, and policy need more development. For example, how might cross-cultural variation explain differences across clusters, and what lessons can practitioners draw when designing anthropomorphic strategies in global contexts? Similarly, the conclusion should clarify boundary conditions by identifying when anthropomorphism strengthens consumer trust and when it risks eliciting scepticism or discomfort.

 The limitations should be discussed in a more direct manner. The restriction to Scopus-indexed, English-language publications risks overrepresenting Western perspectives, which should be acknowledged as a key boundary condition. The authors could also point to the need for multilingual, multi-database reviews to provide a more global and balanced perspective. In addition, clearer directions for future empirical work would strengthen the conclusion—for instance, recommending cross-cultural experiments, longitudinal studies, or sector-specific investigations in tourism, healthcare, and retail.

 Finally, the manuscript would benefit from careful language editing. Some sentences are overly long and certain terms are inconsistently used. A round of stylistic polishing to correct grammar and improve readability will ensure that the manuscript meets professional standards.

**3. Recommendation**

 The manuscript has progressed considerably and offers an informative synthesis of anthropomorphism research. With further revisions—particularly a tighter theoretical linkage, more concise results presentation, and expanded discussion of limitations—the article has strong potential to make a meaningful contribution. I recommend **major revision** at this stage to allow the authors to fully realize the manuscript’s promise.

Are the rationale for, and objectives of, the Systematic Review clearly stated?

Yes

Is the statistical analysis and its interpretation appropriate?

Not applicable

If this is a Living Systematic Review, is the ‘living’ method appropriate and is the search schedule clearly defined and justified? (‘Living Systematic Review’ or a variation of this term should be included in the title.)

Not applicable

Are sufficient details of the methods and analysis provided to allow replication by others?

No

Are the conclusions drawn adequately supported by the results presented in the review?

Partly

Reviewer Expertise:

NA

I confirm that I have read this submission and believe that I have an appropriate level of expertise to confirm that it is of an acceptable scientific standard, however I have significant reservations, as outlined above.

# Reviewer report: Reviewer response for version 1

**DOI:** 10.5256/f1000research.178305.r390874

**Published:** 2025-07-22

**Authors:** Api Adyantari

**Affiliations:** 1 Atma Jaya University Yogyakarta, Yogyakarta, Indonesia

**Strengths of this article:**

- **Timeliness and Relevance**: The focus on anthropomorphism aligns with current trends in digital marketing, AI, and consumer-brand interaction.
- **Comprehensive Scope**: Covers 2010 to 2024 and includes multiple dimensions (citations, keywords, journals, countries).
- **Research clusters and themes: **The six thematic areas, such as AI/service robots, branding, chatbot commerce, tourism, consumer perception, and anthropomorphic interaction, are supported by co-word and co-citation analysis results.
- **Use of Bibliometric Tools**: Employs VOSviewer and Bibliometrix-R, which are standard tools in the field.
- **Structured Presentation**: The article is organized around clear sections, with detailed figures and thematic clustering.

**Major Concerns:**

- **Clarity and Coherence of Argumentation: **The introduction outlines the goals well, but kindly give an early statement summarizing what is novel about this review compared to past reviews (e.g., coverage of specific domains, use of particular tools, thematic clusters). There is noticeable repetition, especially when discussing the benefits and risks of anthropomorphism (e.g., the uncanny valley is cited several times in similar ways).
- **Methodology Clarity: **Need to include the exact Scopus search string and syntax, and need more clarity about the article's screening process. Kindly describe the data cleaning, deduplication, etc.
- **Strengthen critical analysis in thematic sections: **The six cluster themes are informative, but mainly descriptive. Authors can compare and contrast perspectives (e.g., Western vs. Eastern cultural attitudes toward anthropomorphism), and may also explain tensions or gaps in the literature per cluster.
- **Overgeneralized Conclusions**: The conclusion implies strong managerial implications, but few concrete links are made between clusters and real-world business applications (e.g., sector-specific cases in retail, healthcare, etc.). Some concluding statements, such as anthropomorphism being a “transformative strategy” in business and “contributing to daily life,” lack direct support from the reviewed data. Rephrase claims to align with the evidence presented. Add citations to support stronger claims.

**Minor Concerns:**

- There are some minor grammatical issues. Avoid overusing certain phrases. Phrases like “emotional closeness,” “enhances relationship,” and “relevant to technology” are repeated often, suggesting using synonyms to maintain engagement.

**Summary:**

 This article presents a well-structured and timely bibliometric review on anthropomorphism in the domains of business, management, and accounting. Its strengths lie in the comprehensive scope, methodical use of bibliometric tools, and relevance to ongoing developments in AI and digital marketing. The identification of six major thematic clusters adds value to the literature and helps shape a clearer understanding of the evolving academic landscape.

 However, several areas require improvement to enhance the article’s rigor and academic contribution. Key concerns include the need for methodological transparency, especially regarding search strategies, data processing, and inclusion criteria, and for more critical depth in discussing thematic findings. The conclusions, while ambitious, should be more tightly anchored to the presented evidence, and overgeneralized claims need to be revised or substantiated. Finally, improving language consistency and reducing repetition will strengthen the overall clarity and readability.

 With these revisions, the manuscript has the potential to serve as a valuable and credible reference for scholars and practitioners exploring the role of anthropomorphism in modern business contexts.

Are the rationale for, and objectives of, the Systematic Review clearly stated?

Yes

Is the statistical analysis and its interpretation appropriate?

Not applicable

If this is a Living Systematic Review, is the ‘living’ method appropriate and is the search schedule clearly defined and justified? (‘Living Systematic Review’ or a variation of this term should be included in the title.)

Not applicable

Are sufficient details of the methods and analysis provided to allow replication by others?

Yes

Are the conclusions drawn adequately supported by the results presented in the review?

Partly

Reviewer Expertise:

marketing management, marketing, digital marketing, e-commerce

I confirm that I have read this submission and believe that I have an appropriate level of expertise to confirm that it is of an acceptable scientific standard, however I have significant reservations, as outlined above.

# Response

**Published:** 2025-08-19

**Authors:** Diesyana Ajeng Pramesti

**Affiliations:** Management, Universitas Muhammadiyah Magelang, Magelang, Central Java, Indonesia

**Reviewer’s comment : Strengths of this article**

- Timeliness and Relevance: The focus on anthropomorphism aligns with current trends in digital marketing, AI, and consumer-brand interaction.
- Comprehensive Scope: Covers 2010 to 2024 and includes multiple dimensions (citations, keywords, journals, countries).
- Research clusters and themes: The six thematic areas, such as AI/service robots, branding, chatbot commerce, tourism, consumer perception, and anthropomorphic interaction, are supported by co-word and co-citation analysis results.
- Use of Bibliometric Tools: Employs VOSviewer and Bibliometrix-R, which are standard tools in the field.
- Structured Presentation: The article is organized around clear sections, with detailed figures and thematic clustering.

**Author’s responses: Strengths of this article**

 We sincerely thank and appreciate to the reviewer for the positive evaluation of our manuscript.

- We are pleased that the paper’s focus on anthropomorphism is recognized as timely and relevant to ongoing developments in digital marketing, AI, and consumer–brand interaction.
- We set the time limits (2010-2024) and criteria’s (citations, keywords, journals, countries) to obtain specific and robust results, thereby achieving the objectives of this systematic literature review.

- We use Bibliometrix-R and VOSviewer to generate more comprehensive findings through the integration of detailed visual representations and systematically organized thematic groupings.

---------------------------------------------------------------------------------------------------------------------

**Reviewer’s comment :** **Major Concerns**

1. **Clarity and Coherence of Argumentation:**

- The introduction outlines the goals well but kindly give an early statement summarizing what is novel about this review compared to past reviews (e.g., coverage of specific domains, use of tools, thematic clusters).
- There is noticeable repetition, especially when discussing the benefits and risks of anthropomorphism (e.g., the uncanny valley is cited several times in similar ways).

**Author’s responses:**

 We appreciate the reviewer’s comment regarding the need for clearer articulation of the novelty of our study and improved coherence of argumentation and deleted a noticeable repetition. In response,

- We have revised the Introduction by adding one paragraph that **highlights the distinctive contributions **and **several sentences** in paragraph 5 (orange colour) as follows:

This literature review offers a broader and more integrated scope by covering anthropomorphism across multiple applied domains such as branding, retail, service industries, tourism, AI, chatbots, and service robots, while many earlier studies focus on narrower contexts like brand anthropomorphism (Sharma, 2022), hospitality and tourism (Ding, 2022), food marketing (Mishra, 2023), and service robots (Zhang, 2024). To get more comprehensive result, this article employs a dual methodology in bibliometric approach using Bibliometrix-R and VOSviewer to conduct co-word, co-citation, and thematic evolution analyses, complemented by descriptive statistics of citations, keywords, journals, and countries. It is difference with the most prior reviews rely only a single bibliometric tool like PRISMA-based SLRs, or qualitative thematic synthesis without large-scale bibliometric mapping (Sharma & Rahman (2022); Ding et al., (2022); Zhang et al., (2024); Kaifeng & Pengbo (2024); Mishra & Mehta (2023)).

 This study also explicitly linked to key theoretical frameworks such as mind perception theory, trust theory, and symbolic interactionism, whereas previous works tend to group findings into broad conceptual categories without bibliometric-derived or theory-integrated clustering (Brown et al., 2019; Guido & Peluso, 2015; Payne et al., 2013; Tillery & McGill, 2015).

- We have eliminated repeated revision to the same concept which previously appeared with similar phrasing, as follows

- In Introduction section,  paragraph 3 page 2: …”significant risks may arise, such as manipulative perceptions, distrust, and even the "uncanny valley"(Mori et al., 2012) **,** …” **changed to** while offering notable benefits, anthropomorphism also presents potential drawbacks, such as manipulative perceptions and reduced trust including phenomena further discussed on Anthropomorphism in Branding section.”
- In Method section, paragraph 1 page 4: …” without risking bias” **changed to** …” while minimizing potential bias”.
- In Finding and Discussion section part 4.2.3. Co-word network analysis: Mapping impact through emerging keywords, paragraph 2, page 12: after words "tourism," "technology development," and "hospitality industry," **added** “which are discussed in detail in the Anthropomorphism in Tourism cluster section”

- In Finding and Discussion section part 4.4 Promising Research Topics part Consumer Behavior, page 22: after … “uncanny valley” **added a sentence** “a perceptual drop in positive response when features appear almost, but not fully, humanlike (Mori et al., 2012), as seen in certain brand mascots, virtual influencers, and AI avatars.”

- In Finding and Discussion section part 4.4 Promising Research Topics part Technology Adoption, page 22: …” risk perception” **changed to** perceived uncertainty.

---------------------------------------------------------------------------------------------------------------------

1. **Methodology Clarity **

Need to include the exact Scopus search string and syntax, and need more clarity about the article's screening process. Kindly describe the data cleaning, deduplication, etc.

**Author’s responses:**

 Thank you for valuable suggestion. We agree that transparency in the data collection and cleaning process is essential for reproducibility.

- Regarding about the exact Scopus search string and syntax, we would like to confirm that syntax **already** **written** in the text, in part Bibliometric Technique Used in paragraph 2  page 5 and in Figure 1:..”

words related to anthropomorphism, such as "anthropomophism" OR "anthropomorphisms" AND “brand anthropomorphism” AND “perceived anthropomorphism” AND “product anthropomorphism” AND “human like” AND “Marketing” AND “dehumanization” AND “consumer behavior” AND “communication” AND “persuasion”, were used as keywords. The authors then filtered the data and reviewed each relevant article according to the topic of anthropomorphism with several limitations using the formula YEAR-ABS-KEY ( "2010-2024" ) ) AND ( LIMIT-TO ( SUBJAREA, "BUSI" ) ) AND ( LIMIT-TO ( DOCTYPE, "ar" ) ) AND ( LIMIT-TO ( PUBSTAGE, "final" ) ) AND ( LIMIT-TO ( SRCTYPE, "j" ) ) AND ( LIMIT-TO ( LANGUAGE, "English" ) ).”  

- In the revised manuscript, we have added the explanation of PRISMA (Preferred Reporting Items for Systematic Reviews and Meta-Analyses) protocol, which includes identification, screening, eligibility, and inclusion in paragraph 2 line 3 page 4-5, as follows:

The first stage is identification stage, a comprehensive search strategy is implemented across multiple scholarly databases, gray literature sources, and other relevant repositories using predefined keywords to capture all potentially relevant records. Then screening stage, involves the removal of duplicate entries, followed by a preliminary assessment of titles and abstracts to exclude studies that do not meet the broad inclusion criteria. Subsequently, in the eligibility stage, full-text articles of the remaining studies are critically examined against predefined inclusion and exclusion criteria, with reasons for exclusion explicitly documented. The final stage, inclusion, the set of studies that satisfy all eligibility requirements and form the evidence base for data extraction, synthesis, and analysis. This entire process is typically summarized using a PRISMA flow diagram, which presents the number of records at each stage and the rationale for exclusions, thereby enhancing the clarity and traceability of the review methodology (Figure 1). After the final stage, the author still carried out final checking **regarding manual data cleaning which includes duplication**, keyword harmonization, and metadata correction.

 ---------------------------------------------------------------------------------------------------------------------

  

1. **Strengthen critical analysis in thematic sections**

The six cluster themes are informative, but mainly descriptive. Authors can compare perspectives (e.g., Western vs. Eastern cultural attitudes toward anthropomorphism) and may also explain tensions or gaps in the literature per cluster.

**Author’s responses:**

 Thank you for this valuable recommendation. In response, we have revised the discussion of each of the six clusters as follows:

- We added several sentences in the earlier paragraph in page 14 line 6.

These interactions are influenced by consumers' cultural backgrounds. Cultural context significantly shapes attitudes toward anthropomorphism, both in branding strategy, technology, and consumer behavior (Baskentli et al, 2023; Spatola et al, 2022). Culture influences consumer acceptance and perceived effectiveness of human-like design features.

- In every cluster, we are adding explanation with two perspectives of culture.

1. AI and service robotics: Advancing human-robot collaboration (sub section research clusters on anthropomorphism part AI and service robotics: advancing human-robot collaboration, paragraph 2 and 3 page 14-15)

Although research from 2020–2025 shows increasing interest in human-robot collaboration, most studies still focus on technical aspects, efficiency, and general user acceptance. However, there is a gap in cross-cultural understanding of how anthropomorphism influences perceptions of trust and acceptance of service robots across contexts (Gajić et al., 2024).

 In the realm of AI and service robotics, cultural orientation significantly shapes responses to anthropomorphic designs.  In Western contexts, a pragmatic and utilitarian mindset predominates anthropomorphic features are embraced when they enhance usability, task efficiency, and trust, yet designers remain cautious of designs that verge on excessive human likeness due to the "uncanny valley" phenomenon—an effect whereby entities that appear almost, but not entirely, human elicit perceptual discomfort and unease (Gee, F.C. & Browne, et al, 2005; Castelo, N., Sarvary, M, 2022). In contrast, Eastern cultures particularly in Japan, are deeply influenced by animist traditions and philosophies such as Shintoism and Confucian collectivism, which foster a worldview where non-human entities are perceived as animated and integral to social harmony (Spatola et al, 2022). Consequently, humanlike robots are more readily accepted as collaborative partners rather than mere tools, facilitating harmonious human–robot relationships grounded in cultural affinity and relational cognition.

1. Anthropomorphism in branding (sub section research clusters on anthropomorphism part anthropomorphism in branding, paragraph 2 page 15)

Western consumers appreciate anthropomorphic mascots and virtual influencers for their ability to create emotional resonance, yet they tend to guard against brand dilution and manipulative perceptions, prioritizing alignment with individuality and consumer autonomy. Eastern consumers, by contrast, often interpret humanlike brand imagery as a sign of approachability and communal harmony, with anthropomorphic branding strengthening long-term loyalty and trust in line with collectivist social norms.

1. Anthropomorphic AI interactions (sub section research clusters on anthropomorphism part anthropomorphism in AI interactions, paragraph 3 and 3 page 16)

Western audiences generally accept human-like AI when it serves functional and experiential purposes. However, they may be uncomfortable with excessive emotionality, citing privacy and ethical concerns. However, Eastern audiences tend to be more comfortable with emotionally expressive AI, viewing it as a legitimate social actor that facilitates relational engagement, reflecting a cultural openness to the integration of human and non-human agency. Anthropomorphized AI interactions have been widely researched in the context of chatbots, voice assistants, and recommendation systems. However, significant gaps exist in understanding the long-term psychological impact of these interactions, particularly regarding dependency, blind trust, or potential consumer manipulation. Most studies are still short-term experimental (Zheng et al., 2022).

1. Anthropomorphism and consumer perception (sub section research clusters on anthropomorphism part anthropomorphism and consumer perception, paragraph 2 page 17)

Given the perceptions of Western consumers, who often evaluate anthropomorphic cues based on their impact on autonomy and authenticity, excessive use risks skepticism or perceptions of manipulation. Such perceptions may lead consumers to question the credibility of the brand, thereby weakening trust and reducing long-term engagement (Goel, P., & Garg, A., 2025). Contrast to Eastern consumer perceptions, such gestures are generally viewed as genuine attempts to build trust and harmony, in line with cultural expectations of warmth, friendliness, and caring from brands and products. Recent research has confirmed that anthropomorphism can enhance trust, emotional intimacy, and consumer loyalty. However, the psychological mechanisms that differ across consumer segments (e.g., age, gender, and culture) remain underexplored. Some studies only address general effects without distinguishing perception dynamics in emerging markets (Lee & Lalwani, 2024).

1. Anthropomorphism in tourism (sub section research clusters on anthropomorphism part anthropomorphism in tourism, paragraph 3 page 17-18)

Western markets often use anthropomorphic storytelling in ecotourism or heritage promotion, but emphasize realism, accuracy, and cultural sensitivity to avoid undermining local traditions. Eastern tourism strategies, on the other hand, embrace anthropomorphic characters—such as animal mascots or mythological figures—as an integral part of conveying cultural values, hospitality, and emotional connection to the destination, rooted in a long-standing tradition of personified narratives. The use of anthropomorphism in tourism and hospitality destinations (e.g., robot receptionists or hotel chatbots) is beginning to be explored, but research remains limited to technical issues and service satisfaction, with little attention paid to the emotional and cultural dimensions of tourists. The lack of cross-national comparative studies also highlights a gap in understanding whether anthropomorphism is more effective in collectivist (Eastern) versus individualist (Western) cultures (Gajić et al., 2024). This distinction is crucial when employing anthropomorphic strategies in tourism development.

1. Chatbots in commerce (sub section research clusters on anthropomorphism part chatbots in commerce, paragraph 1 and 2 page 18)

However, when using chatbots in communication with humans, consideration must also be given to the consumer's background. Western consumers focus on speed, accuracy, and problem-solving abilities, welcoming anthropomorphic features such as names or avatars only if they enhance the user experience without sacrificing efficiency. Eastern consumers often respond to chatbots with friendly, human-like personalities that adhere to norms of interpersonal politeness, using anthropomorphic designs to build rapport, reduce perceived social distance, and encourage repeat interactions.

 However, it is also important to consider and understand the negative effects such as over trust, perceived manipulation, and consumer fatigue that result from intensive interactions with chatbots. There is still limited research linking chatbot anthropomorphism with long-term purchasing behavior and the sustainability of customer relationships (Fan et al., 2024), so the effectiveness of anthropomorphic chatbots in increasing customer satisfaction remains questionable.

 ---------------------------------------------------------------------------------------------------------------------

1. **Overgeneralized Conclusions**

The conclusion implies strong managerial implications, but few concrete links are made between clusters and real-world business applications (e.g., sector-specific cases in retail, healthcare, etc.). Some concluding statements, such as anthropomorphism being a “transformative strategy” in business and “contributing to daily life,” lack direct support from the reviewed data. Rephrase claims to align with the evidence presented. Add citations to support stronger claims.

**Author’s responses:**

 We thank the reviewer for highlighting the need to align our conclusions more closely with the reviewed data and to ground our managerial implications in real-world examples. We try to rewrite the conclusion according to the directions.

**Reviewer’s comment :** **Minor Concerns**

1. **Reviewer’s comment :**

There are some minor grammatical issues. Avoid overusing certain phrases. Phrases like “emotional closeness,” “enhances relationship,” and “relevant to technology” are repeated often, suggesting using synonyms to maintain engagement.

**Authors’s response:**

 Thank you for your valuable feedback regarding the language and grammar. We have carefully revised the manuscript by removing repetitive phrases and replacing them with alternative expressions to improve readability, while ensuring that the original meaning and intended arguments remain unchanged. We only used Frase “emotional closeness” only in Literature Review, paragraph 2 line 5 page 4 .  We changed others to “emotional attachment”  in sub section Research Clusters on Anthropomorphism, sub-sub section Anthropomorphism and consumer perception, paragraph 1 line 5 page 17 and “emotional intimacy” in paragraph 2 line 8 page 17

 ---------------------------------------------------------------------------------------------------------------------

**Addition References:**

  

1. Spatola, N., Marchesi, S., & Wykowska, A. (2022). The personality of anthropomorphism: How the need for cognition and the need for closure define attitudes and anthropomorphic attributions toward robots. Frontiers in Robotics and AI, 9, 863319. https://doi.org/10.3389/frobt.2022.863319
2. Castelo, N., Sarvary, M (2022). Cross-Cultural Differences in Comfort with Humanlike Robots. Int J of Soc Robotics 14, 1865–1873. https://doi.org/10.1007/s12369-022-00920-y
3. Fan, W., Osman, S., Zainudin, N., & Yao, P. (2024). How information and communication overload affect consumers’ platform switching behavior in social commerce. *Heliyon, 10*(10).
4. Gajić, T., Vukolić, D., Bugarčić, J., Đoković, F., Spasojević, A., Knežević, S., ... & Dávid, L. D. (2024). The adoption of artificial intelligence in Serbian hospitality: A potential path to sustainable practice. *Sustainability, 16*(8), 3172.
5. Gee, F.C. & Browne, Will & Kawamura, Kazuhiko. (2005). Uncanny valley revisited. 151 - 157. 10.1109/ROMAN.2005.1513772.
6. Kim, J. J. (2024). Brand portfolio extension of international hotel chains: A perspective on consumer confusion and consumer decision-making process. *International Journal of Contemporary Hospitality Management, 36*(9), 3093–3111.
7. Lee, H., & Lalwani, A. (2023). Power Distance Belief and Consumer Purchase Avoidance: Exploring the Role of Cultural Factors in Retail Dynamics. Journal of Marketing Research, 61, 349 - 367. https://doi.org/10.1177/00222437231182600.
8. Mori, M., MacDorman, K., & Kageki, N. (2012). The Uncanny Valley [From the Field]. IEEE Robotics & Automation Magazine, 19, 98–100. https://doi.org/10.1109/MRA.2012.2192811
9. Zheng, H., & Jiang, S. (2022). Linking the pathway from exposure to online vaccine information to cyberchondria during the COVID-19 pandemic: A moderated mediation model. *Cyberpsychology, Behavior, and Social Networking, 25*(10), 625–633.
10. Ding, A., Lee, R. H., Legendre, T. S., & Madera, J. (2022). Anthropomorphism in hospitality and tourism: A systematic review and agenda for future research. *Journal of Hospitality and Tourism Management*, *52*, 404–415. https://doi.org/10.1016/j.jhtm.2022.07.018
11. Kaifeng, L., & Pengbo, S. (2024). Effectiveness of facial anthropomorphism design for improving multimedia learning outcomes: Systematic review and meta-analysis. *Smart Learning Environments*, *11*(1), 42. https://doi.org/10.1186/s40561-024-00332-7
12. Mishra, R., & Mehta, R. (2023). The effects of food anthropomorphism on consumer behavior: A systematic literature review with integrative framework and future research directions. *Appetite*, *190*, 107035. https://doi.org/10.1016/j.appet.2023.107035
13. Brown, J. R., Crosno, J. L., & Tong, P. Y. (2019). Is the theory of trust and commitment in marketing relationships incomplete? Industrial Marketing Management, 77, 155–169. https://doi.org/10.1016/j.indmarman.2018.10.005
14. Tillery, M., & McGill, A. L. (2015). Who or What to Believe: Trust and the Differential Persuasiveness of Human and Anthropomorphized Messengers. Journal of Marketing, 79(4), 94–110. https://doi.org/10.1509/jm.12.0166
15. Goel, P., & Garg, A. (2025). Virtual personalities, real bonds: anthropomorphised virtual influencers’ impact on trust and engagement. Journal of Consumer Marketing. https://doi.org/10.1108/jcm-05-2024-6915.

# Reviewer report: Reviewer response for version 1

**DOI:** 10.5256/f1000research.178305.r393191

**Published:** 2025-07-22

**Authors:** Taeshik Gong

**Affiliations:** 1 Hanyang University, Ansan, South Korea

The submitted manuscript offers a comprehensive bibliometric review of anthropomorphism research in business, management, and accounting from 2010 to 2024, using Bibliometric-R and VOSviewer tools. The authors aim to map research trends, identify influential contributions, and propose future directions. The scope of the paper is timely and relevant, especially given the increasing interest in AI-human interaction and service technologies. The manuscript is informative and ambitious in scale, and it delivers a substantial compilation of existing literature. However, despite its merits, the paper suffers from a number of critical conceptual, methodological, and structural issues that require significant attention before it can be considered for indexing.

 1. Strengths

 The manuscript addresses an important and underexplored gap by synthesizing the state of anthropomorphism research across business disciplines. The use of PRISMA, Bibliometric-R, and VOSviewer is appropriate, and the scale of the dataset (326 articles from Scopus) lends credibility to the mapping effort. The visualization of citation, co-citation, co-word, and co-authorship networks is particularly helpful. The paper also makes a commendable effort to delineate clusters and research streams such as AI and service robots, branding, and chatbot design.

 2. Major Concerns

 (1) Conceptual Framing and Contribution

 The manuscript frequently conflates description with theorization. Although the authors succeed in cataloguing trends and summarizing thematic domains, there is little effort to develop an overarching conceptual framework or meta-theoretical synthesis. For a paper that aims to “unveil” anthropomorphism in business, it lacks a critical engagement with underlying assumptions, tensions, or divergent paradigms in the field (e.g., psychological vs. sociocultural interpretations of anthropomorphism). A stronger analytical lens is needed to move beyond summary toward explanation.

 (2) Limited Integration of Theories

 While the paper references foundational works (e.g., Epley et al., Aggarwal & McGill), it does not offer an integrative account of how anthropomorphism has been theorized or operationalized across subfields. For instance, the Technology Acceptance Model (TAM), UTAUT, social cognition theory, and affective-motivational theories are mentioned in passing but not systematically discussed. There is no analysis of how theoretical paradigms have shifted over time, nor of disciplinary boundaries (e.g., marketing vs. hospitality vs. IS).

 (3) Methodological Transparency and Rigor

 Although PRISMA is mentioned, the methodological section lacks clarity and replicability. Key details are missing regarding:

 -How articles were coded into specific themes or clusters

 -How inter-coder reliability (if any) was established

 -Whether full-texts were analyzed or only abstracts and keywords

 -How keywords were refined for co-word analysis (e.g., stemming, synonym merging)

 Without methodological rigor, the claims about the dominance or evolution of themes risk being anecdotal rather than empirical.

 (4) Overreliance on Descriptive Analysis

 Much of the discussion section is taken up by numerical summaries and frequency counts (e.g., publication trends by year, author country, journal ranking). While such descriptive statistics are valuable, they dominate at the expense of analytical depth. The manuscript would benefit from including more concrete interpretive insights into why certain themes have emerged or declined (e.g., impact of COVID-19, rise of chatbot ethics, cultural variations in consumer AI acceptance).

 (5) Language, Structure, and Redundancy

 The writing is generally readable but repetitive and occasionally imprecise. Several sentences are vague or inflated (e.g., “This paper plays an important role and contributes significantly to the development of business and daily life”). Some sections, such as the repetition of results in the discussion, could be significantly condensed. A tighter structure around clearly framed research questions and thematic insights would greatly improve coherence.

 (6) Superficial Treatment of Limitations and Ethical Issues

 The limitations section acknowledges the narrow temporal and linguistic scope, but other concerns remain unaddressed. For example, what are the epistemological limits of bibliometric tools in studying complex constructs like anthropomorphism? What ethical challenges arise in applying anthropomorphism in commercial AI systems (e.g., manipulation, surveillance, consent)? These dimensions deserve at least a brief critical reflection, especially in the conclusion.

 (7) Recommendations for Revision

 -Reorganize the manuscript around clearer research questions and thematic narratives.

 -Develop a conceptual model or framework to integrate findings across clusters.

 -Strengthen the methodological section with detailed protocols and coding logic.

 -Deepen the theoretical engagement across domains and over time.

 -Reduce descriptive redundancy and aim for analytical richness.

 -Expand the discussion of limitations, cultural-contextual variation, and ethical implications.

 3. Conclusion

 This manuscript has potential to make a valuable contribution as a foundational bibliometric review of anthropomorphism in business disciplines. However, its current form remains too descriptive and lacks the theoretical and methodological depth expected of a scholarly systematic review. Major revisions are necessary to enhance its analytical sophistication, theoretical contribution, and narrative coherence. I encourage the authors to address the above issues and resubmit a significantly revised version.

Are the rationale for, and objectives of, the Systematic Review clearly stated?

Yes

Is the statistical analysis and its interpretation appropriate?

Not applicable

If this is a Living Systematic Review, is the ‘living’ method appropriate and is the search schedule clearly defined and justified? (‘Living Systematic Review’ or a variation of this term should be included in the title.)

Not applicable

Are sufficient details of the methods and analysis provided to allow replication by others?

No

Are the conclusions drawn adequately supported by the results presented in the review?

Partly

Reviewer Expertise:

Service failure and recovery, customer justice perceptions, and forgivenessHuman–robot interaction in hospitality and retail contextsAI and service automation impacts on consumer behavior and employee adaptationBrand attachment, nostalgia, and self-concept in consumer–brand relationshipsCognitive and emotional processes in consumer decision-making, including envy, empathy, and role consistencyAlgorithmic management and its dual impact on gig worker well-being (job demands/resources)Fairness, trust, and interaction design in technologically mediated service encounters

I confirm that I have read this submission and believe that I have an appropriate level of expertise to state that I do not consider it to be of an acceptable scientific standard, for reasons outlined above.
